# Enzymatic study on AtCCD4 and AtCCD7 and their potential to form acyclic regulatory metabolites

**DOI:** 10.1093/jxb/erw356

**Published:** 2016-10-06

**Authors:** Mark Bruno, Julian Koschmieder, Florian Wuest, Patrick Schaub, Mirjam Fehling-Kaschek, Jens Timmer, Peter Beyer, Salim Al-Babili

**Affiliations:** ^1^Albert-Ludwigs University of Freiburg, Faculty of Biology, Schaenzlestr. 1, D-79104 Freiburg, Germany; ^2^Albert-Ludwigs University of Freiburg, Department of Physics, Hermann-Herder-Str. 3a, D-79104 Freiburg, Germany; ^3^Albert-Ludwigs University of Freiburg, BIOSS Center for Biological Signalling Studies, Schaenzlestr. 18, D-79104 Freiburg, Germany; ^4^King Abdullah University of Science and Technology (KAUST), BESE Division, Center for Desert Agriculture, 23955-6900 Thuwal, Saudi Arabia

**Keywords:** Apocarotenoids, carotenoids, carotenoid cleavage dioxygenase, CCD4, CCD7, retrograde signaling

## Abstract

The Arabidopsis carotenoid cleavage dioxygenase 4 (AtCCD4) is a negative regulator of the carotenoid content of seeds and has recently been suggested as a candidate for the generation of retrograde signals that are thought to derive from the cleavage of poly-*cis*-configured carotene desaturation intermediates. In this work, we investigated the activity of AtCCD4 *in vitro* and used dynamic modeling to determine its substrate preference. Our results document strict regional specificity for cleavage at the C9–C10 double bond in carotenoids and apocarotenoids, with preference for carotenoid substrates and an obstructing effect on hydroxyl functions, and demonstrate the specificity for all-*trans*-configured carotenes and xanthophylls. AtCCD4 cleaved substrates with at least one ionone ring and did not convert acyclic carotene desaturation intermediates, independent of their isomeric states. These results do not support a direct involvement of AtCCD4 in generating the supposed regulatory metabolites. In contrast, the strigolactone biosynthetic enzyme AtCCD7 converted 9-*cis*-configured acyclic carotenes, such as 9-*cis*-ζ-carotene, 9'-*cis*-neurosporene, and 9-*cis*-lycopene, yielding 9-*cis*-configured products and indicating that AtCCD7, rather than AtCCD4, is the candidate for forming acyclic retrograde signals.

## Highlight

*In vitro* study shows that AtCCD4 claves all-*trans*-bicyclic-carotenoids, excludes its direct involvement in generating plastid retrograde signals supposedly derived from *cis*-desaturation intermediates, and demonstrates that AtCCD7 converts 9-*cis*-acylic carotenes.

## Introduction

**Fig. 1.**
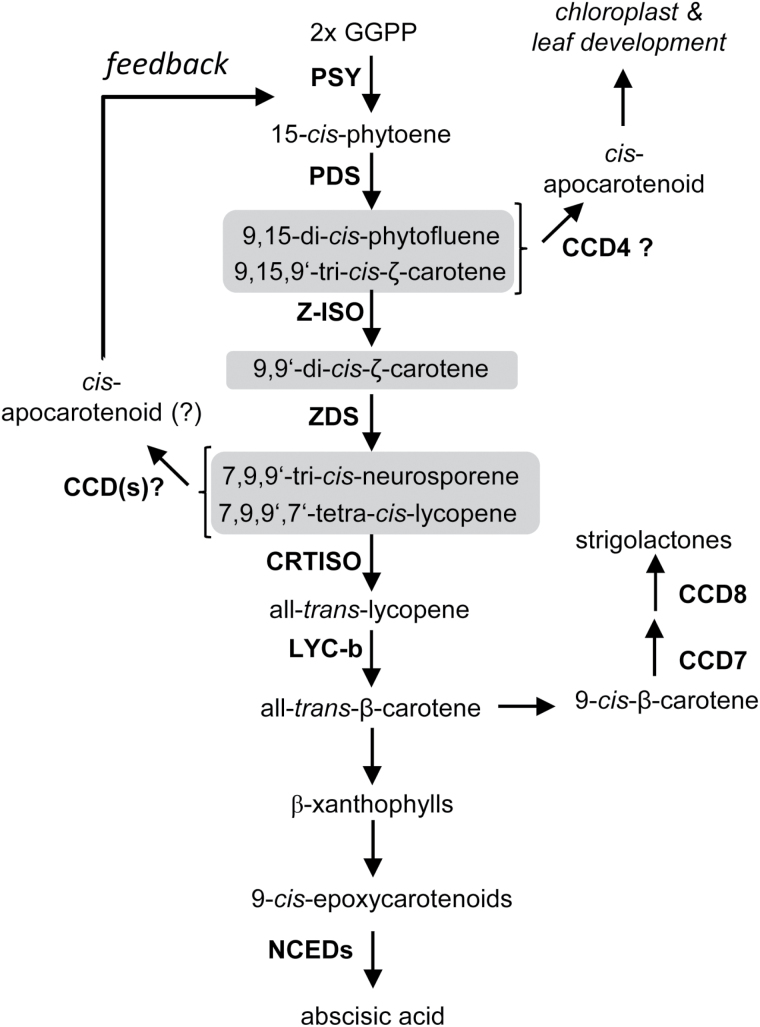
Carotenoid biosynthesis and formation of carotenoid-derived signaling molecules. Desaturation intermediates are shaded. The scheme shows established and hypothetical signals generated by carotenoid cleavage dioxygenases (CCDs). The formation of abscisic acid and SLs is initiated by 9-*cis*-epoxycarotenoid cleavage dioxygenases (NCEDs) and CCD7, respectively. Hypothetical signals are indicated by a question mark. PSY, phytoene synthase; PDS, phytoene desaturase; Z-ISO, ζ-carotene isomerase; CRTISO, carotenoid *cis*-*trans* isomerase; LCY-b, lycopene β-cyclase; D27 (DWARF27), all-*trans*/9-*cis*-β-carotene isomerase. See text for further explanations.

Apocarotenoids are carotenoid cleavage products. They are generally synthesized by members of the carotenoid cleavage oxygenase (CCO) family, which are present in animals, plants, fungi, and eubacteria (von Lintig and Vogt, 2000; Ruch *et al.*, 2005; Auldridge *et al.*, 2006; Prado-Cabrero *et al.*, 2007; Walter and Strack, 2011; Sui *et al.*, 2013; Nisar *et al.*, 2015). Whether these enzymes utilize a monooxygenase or a dioxygenase mechanism remains debated; however, the plant enzymes are commonly referred to as carotenoid dioxygenases (CCDs). The formation of apocarotenoids in plants can be the starting point for biosynthetic pathway branches leading to important regulatory compounds (Fig. 1), such as the phytohormones abscisic acid (ABA) (Schwartz *et al.*, 1997; Zhu, 2002; Seo, 2002) and the strigolactones (SLs; Alder *et al.*, 2012; Al-Babili and Bouwmeester, 2015).

The Arabidopsis CCD family includes nine enzymes classified as either 9-*cis*-epoxycarotenoid dioxygenases (NCEDs), NCED2, 3, 5, 6, and 9, or CCDs, CCD1, 4, 7, and 8. NCEDs are involved in ABA biosynthesis and catalyze the stereospecific cleavage of the C11–C12 double bond in 9-*cis*-configured epoxycarotenoids, to yield the ABA precursor xanthoxin (Schwartz *et al.*, 1997). CCD1 is the only plant CCD that is localized in the cytosol. Arabidopsis AtCCD1 (Schwartz *et al.*, 2001) and homologs from several plant species catalyze symmetrical double cleavage at the C9–C10 and C9'–C10' double bonds in cyclic and acyclic all-*trans*-configured carotenoids. CCD1 enzymes also target other double bonds in linear carotenes, yielding flavors, such as 6-methyl-5-hepten-2-one, geranial, and farnesyl acetone (Vogel *et al.*, 2008; Ilg *et al.*, 2009, 2014). Additional cleavage sites in apocarotenoids have also been documented (Ilg *et al.*, 2014). Moreover, it was assumed that CCD1-type enzymes convert apocarotenoids rather than carotenoids *in planta* and that they may act as a scavenger for oxidatively damaged carotenoids (Scherzinger and Al-Babili, 2008; Ilg *et al.*, 2010). In contrast, CCD7 and CCD8 are devoted to the biosynthesis of SLs (Gomez-Roldan *et al.*, 2008; Umehara *et al.*, 2008; Al-Babili and Bouwmeester, 2015). Here, CCD7 catalyzes the stereospecific cleavage of the *trans*-configured C9'–C10' double bond in 9-*cis*-β-carotene produced by the all-*trans*/9-*cis*-β-carotene isomerase DWARF27 (Alder *et al.*, 2012; Bruno and Al-Babili, 2016), yielding β-ionone and 9-*cis*-β-apo-10'-carotenal (Alder *et al.*, 2012; Bruno *et al.*, 2014). The latter is then converted by CCD8, which catalyzes combination of repeated oxygenation and intramolecular rearrangements, into carlactone (Alder *et al.*, 2012). However, CCD8 enzymes also catalyze a common CCD reaction by cleaving all-*trans*-β-apo-10'-carotenal/ol (Alder *et al.*, 2008) at the C13–C14 double bond, yielding β-apo-13-carotenone (d'orenone). D'orenone is a natural metabolite that can inhibit root hair growth by interfering with auxin transport (Schlicht *et al.*, 2008).

CCD4 enzymes, the subject of this study, regulate carotenoid homeostasis in different tissues, such as Arabidopsis seeds (Gonzalez-Jorge *et al.*, 2013), chrysanthemum petals (Ohmiya *et al.*, 2006), and peach fruit pulp (Brandi *et al.*, 2011; Falchi *et al.*, 2013). CCD4 enzymes from *Crocus*, *Chrysanthemum*, and apple produce β-ionone, indicating a single or double cleavage activity at the C9–C10 and/or the C9'–C10' double bond in β-carotene (Rubio *et al.*, 2008; Huang *et al.*, 2009). It was recently demonstrated that the potato CCD4b cleaves the C9–C10 and/or the C9'–C10' double bond in β-carotene (C_40_), by identifying all-*trans*-β-apo-10'-carotenal (C_27_) as the second product formed together with β-ionone (C_13_) (Bruno *et al.*, 2015). Though with much lower efficiency, the potato enzyme also cleaved α-carotene and unepoxydated xanthophylls, pointing to β-carotene as the main substrate of CCD4 *in planta* (Bruno *et al.*, 2015). However, other members of the CCD4 subfamily show different regional and substrate specificity. For instance, *Citrus* CCD4 enzymes cleave the C7'–C8' double bond in β-carotene, β,β-cryptoxanthin, and zeaxanthin, leading to the fruit peel pigments, β-apo-8'-carotenal and β-citraurin, and the volatiles, cyclocitral and 3-OH-cyclocitral (Rodrigo *et al.*, 2013; Ma *et al.*, 2013).

CCD enzymes might also form hitherto unknown regulatory compounds. Apart from ABA and SLs, additional carotenoid-derived regulatory molecules have been postulated and thought to arise through cleavage of desaturation intermediates of the carotenoid biosynthesis pathway (Fig. 1) (Moise *et al.*, 2014). For instance, the tomato *tangerine* mutant is impaired in the activity of carotenoid *cis*-*trans* isomerase (CRTISO; Fig. 1), which results in 7,9,7',9'-tetra-*cis*-lycopene (prolycopene) accumulation in fruits, while the *r(2997) yellow-flesh* does not express the fruit-specific phytoene synthase gene (PSY1), leading to a lack of fruit carotenoids. It was shown that the accumulation of prolycopene and its poly-*cis*-configured precursors in the *yellow-flesh* background is accompanied by a partial restoration of *PSY1* transcription. This epistatic behavior of the downstream over the upstream pathway activity is thought to be mediated by carotene cleavage product(s) derived from prolycopene or its less desaturated precursors (Kachanovsky *et al.*, 2012). Moreover, the use of Arabidopsis mutants and carotene desaturation inhibitors pointed to the presence of apocarotenoid(s) that modulates leaf and chloroplast development and which is thought to derive from phytofluene and/or ζ-carotene. It has been suggested that AtCCD4 is involved in the formation of this regulatory signal(s) (Avendaño-Vázquez *et al.*, 2014). Furthermore, potato *ccd4* knock-down lines develop tubers with elevated carotenoid content, which also show changed shape and premature sprouting (Campbell *et al.*, 2010). In saffron, *CCD4* transcript levels increase under abiotic stress and during senescence (Rubio *et al.*, 2008; Rubio-Moraga *et al.*, 2014).

In the current work, we investigated the cleavage activity of AtCCD4 *in vitro*, using analytical and kinetic approaches. In addition, we evaluated the potential of this enzyme in generating the signals proposed to derive from *cis*-configured carotene desaturation intermediates, in comparison with the homolog, SL biosynthetic enzyme AtCCD7. Our data indicate that AtCCD7 rather than AtCCD4 may be a candidate capable of forming these signal molecule(s).

## Materials and methods

### Cloning and *in vitro* assays

pThio-AtCCD4: RNA was isolated from 4-week-old Arabidopsis seedlings using Plant RNA Purification Reagent (Invitrogen^®^) and cDNA was synthesized using SuperScript™ RNase H reverse transcriptase (Invitrogen, Paisley, UK). *AtCCD4* full-length cDNA was amplified by PCR with the primers FP 5'-CCGGAGCTCCGGTTATGCCTAACGTG-3' and RP 5'-AGTGAGCTCTATATTGTTAAAGCTTATTAAGGT-3', both containing *Sac*I restriction sites. The digested and purified PCR product was ligated into *Sac*I-digested and dephosphorylated pThio^®^-vector. The pThio-AtCCD7 was described previously (Alder *et al.*, 2012).

pThio-AtCCD4 was transformed into BL21 (DE3) *Escherichia coli* cells harboring the plasmid pGro7 (Takara Bio Inc.). Cells were grown at 37 °C in 50 ml of 2 YT growth medium supplemented with chloramphenicol and ampicillin. Protein expression was induced with 0.2% (w/v) arabinose at an OD_600_ of 0.5. Cells were grown for 4 h at 28 °C and harvested. Cell pellets were re-suspended in 1 ml of modified LEW buffer (50 mM NaH_2_PO_4_, 300 mM NaCl, 1 mg ml^−1^ lysozyme, 1 mM dithiothreitol; pH 8.0), passed twice through a French pressure cell at 10 000 psi, and centrifuged at 20 000 *g* for 5 min. Protein was quantified using the Quick Start™ Bradford Protein Assay (Bio-Rad Laboratories) and adjusted to 20 µg µl^−1^.

Assays were performed in a total volume of 200 µl. Purified substrates (30 µM in CHCl_3_) were mixed with 20 µl of ethanolic Triton X-100 (2%, v/v; Sigma), dried using a vacuum evaporator, and dissolved in 100 µl of 2× incubation buffer [2 mM TCEP, 0.4 mM FeSO_4_, 200 mM HEPES-NaOH pH 7.8, and 2 mg ml^−1^ catalase (Sigma)]. Assays were started by the addition of each 50 µl lysate and H_2_O, and then incubated for 1 h, if not stated otherwise, under shaking (200 rpm) at 30 °C in darkness. For extraction, 2 vols of acetone were added, followed by a short sonication and the addition of 3 vols of light petroleum/diethylether (2:1, v/v). After centrifugation, the epiphase was dried and redissolved in 40 µl of CHCl_3_. A 5 µl aliquot of the extract was subjected to HPLC analysis with system 1, using tocopherol acetate (0.1 mg ml^−1^) as an internal standard.

### Substrate preparation and identification

Substrates were purified using thin-layer silica gel plates (Merck). Synthetic apolycopenals and apocarotenals were kindly provided by BASF (Germany). β-Carotene was obtained from Roth, β,β-cryptoxanthin was from Sigma, and neoxanthin, violaxanthin, and α-carotene were from CaroteNature. Lutein was isolated from *Narcissus pseudonarcissus* petals, and 9-*cis*-violaxanthin from fresh spinach leaves. Zeaxanthin, lycopene, and ζ-carotene isomers were extracted from *E. coli* strains expressing the respectively mutagenized carotenoid gene cluster from *Pantoea ananatis* (Prado-Cabrero *et al.*, 2007). 9,15-di-*cis*-Phytofluene, prolycopene, and proneurosporene were isolated from fruits of the *tangerine* tomato mutant and identified with the aid of published data (Clough and Pattenden, 1979; Carey *et al.*, 1983). 9'-*cis*-Phytofluene was isolated from *Dunaliella salina*. 15-*cis*- and all-*trans*-Phytofluene were purchased from CaroteNature. 9-*cis*-β-Carotene was obtained by iodine-isomerization of β-carotene (Zechmeister, 1962) and subsequent HPLC purification. ζ-Carotene isomers were purified using a YMC C_30_ column and MeOH:TBME (3:1, v/v) as the eluent (HPLC system 4). The identification of isomers was carried out according to Carey *et al.* (1983) and Breitenbach and Sandmann (2005). All-*trans*-neurosporene was obtained from *Rhodovulum sulfidophilum* (Hagemann *et al.*, 1996). 9'-*cis*-Neurosporene was prepared enzymatically by CRTI according to Yu *et al.* (2011) and using the substrate 7,9,9'-tri-*cis*-neurosporene isolated from *tangerine* fruit (Carey, *et al.*, 1983). Synthetic 9-*cis*-lycopene was obtained from Buchem (The Netherlands). Carotenoid substrates were quantified photometrically according to their molar extinction coefficients (Britton *et al*, 1995).

### Dynamic modeling and data processing

The decay of β-carotene (β), β-cryptoxanthin (cry), and zeaxanthin (zea) to β-ionone (β-io) and 3-OH-β-ionone (OH-β-io) via the intermediates β-apo-10-carotenal (β-10) and 3-OH-β-apo-10-carotenal (OH-β-10 is modeled by a set of ordinary differential equations (ODEs) following mass-action kinetics:

$$\begin{array}{l} \;\frac{d}{dt}\left[\beta \right]\;=\;-k_{\beta }\cdot \left[\beta \right]\\ \;\frac{d}{dt}\left[cry\right]\;=\;-k_{cry-OH}\cdot \left[cry\right]-k_{cry-\beta }\cdot \left[cry\right]\\ \;\frac{d}{dt}\left[zea\right]\;=\;-k_{zea}\cdot \left[zea\right]\\ \;\frac{d}{dt}\left[\beta -10\right]\;=\;k_{\beta }\cdot \left[\beta \right]+k_{cry-OH}\cdot \left[cry\right]-k_{\beta -10}\cdot \left[\beta -10\right]\\ \;\frac{d}{dt}\left[OH-\beta -10\right]\;=\;k_{cry-\beta }\cdot \left[cry\right]+k_{zea}\cdot \left[zea\right]-k_{OH-\beta -10}\cdot \left[OH-\beta -10\right]\\ \;\frac{d}{dt}\left[\beta -io\right]\;=\;k_{\beta }\cdot \left[\beta \right]+k_{cry-\beta }\cdot \left[cry\right]+k_{\beta -10}\cdot \left[\beta -10\right]\\ \;\frac{d}{dt}\left[OH-\beta -io\right]\;=\;k_{cry-OH}\cdot \left[cry\right]+k_{zea}\cdot \left[zea\right]+k_{OH-\beta -10}\cdot \left[OH-\beta -10\right] \end{array}$$

In total, the model contains 13 free parameters, denoted by θ, which are determined from data. The parameters of interest are the rate constants *k*_*i*_, *i* = β, cry-OH, cry-β, zea, β-10,OH- β-10. Other parameters are the initial concentrations of the states at time point zero and one conversion factor needed to account for a general change in the enzyme activity between datasets shown in Fig. 3A–C).

The parameters θ are estimated via the maximum likelihood method assuming normally distributed noise. The maximization of the likelihood translates into minimizing the cost function

$$\chi^{2}\left(\theta \right)=\underset{i}{\sum }\frac{{\left(x_{i}-x\left(t_{i},\theta \right)\right)}^{2}}{\sigma_{i}^{2}}$$

where *x*_*i*_ denotes the measured value of data point *i* with uncertainty σ_*i*_, and *x*(*t*_*i*_, θ) gives the model prediction at time *t*_*i*_. The ODEs together with their sensitivity equations are integrated with the lsodes solver (Soetaert *et al.*, 2010). Non-linear derivative-based optimization of the cost function is performed by a trust region optimizer (Nocedal and Wright, 1999). Parameter identifiability and confidence intervals are computed by the profile likelihood method (Raue *et al.*, 2009). All analyses have been performed with the packages cOde/dMod for dynamic modeling in R. The detailed model specification encompassing alle experimental conditions can be found in Supplementary dataset S1 at *JXB* online.

#### Data pre-processing

Each experiment was repeated at least three times; some were repeated four times. Mean values of the experiments were taken as input for the modeling. Measurement uncertainties were estimated by the error model $\sigma^{2}\left(x\right)=s_{0}^{2}+s_{\text{rel}}^{2}x^{2}$ which accounts for an absolute and a relative contribution. The variance parameters *s*_0_ and *s*_rel_ are determined via maximum-likelihood estimation from the negative log-likelihood:

$$l\left(s_{0},s_{rel}\right)=\underset{i}{\sum }\frac{1}{2}\text{log}\left(s_{0}^{2}+s_{\text{rel}}^{2}x^{2}\right)-\text{log}\left(\phi_{n_{i}-1}\left(\frac{\left(n_{i}-1\right)\cdot v_{i}}{s_{0}^{2}+s_{\text{rel}}^{2}x^{2}}\right)\right)$$

where ϕ_*ni*_–1 is the probability density of the χ^2^ distribution with *n*_*i*_–1 degrees of freedom and the index corresponds to data point *i* with mean value *x*_*i*_ and variance *v*_*i*_ determined from *n*_*i*_ replicates. Standard errors of the mean were computed from the estimated variance and the number of replicates $\frac{\sigma \left(x_{i}\right)}{\sqrt{n_{i}}}$.

### HPLC analysis and purification

Carotenoids were analyzed using a Shimadzu UFLC XR separation module equipped with an SPD-M20A photodiode array detector (Shimadzu). The column temperature was held at 40 °C. A YMC-pack-C_30_ reversed phase column (150 × 3 mm i.d., 5 µm; YMC Europe) was used throughout. The separation system 1 solvent system consisted of A: MeOH/TBME (1:1, v/v) and B: MeOH/H_2_O/TBME (30:10:1, v/v/v). The flow rate was 0.6 ml min^−1^ with a gradient from 100% B to 0% B within 20 min and maintenance of the final conditions for 4 min. The separation system 2 solvent system utilized A: MeOH/TBME (1:1, v/v) and B: MeOH/H_2_O/TBME (30:10:1, v/v/v). The flow rate was 0.6 ml min^−1^ with a gradient from 100% B to 0% B within 24 min and maintenance of the final conditions for 4 min. Separation system 3 employed the solvent system A: MeOH/TBME (4:1, v/v) and B: MeOH/H_2_O/TBME (30:10:1, v/v/v). The flow rate was 0.6 ml min^−1^ with a gradient from 100% B to 40% B within 20 min, to 0% in 5 min and maintenance of the final conditions for 10 min. Separation system 4 was isocratic with the solvent MeOH/TBME (3:1, v/v) at a flow rate of 2 ml min^−1^.

### Mass spectrometry

Volatile cleavage products such as β-ionone, 6-methyl-5-hepten-2-one (MHO), and geranylacetone were collected by solid phase micro extraction (SPME; PDMS, 100 µm; Supelco). The fiber was exposed to the assay gas phase for 15 min and desorbed in the injector of the Trace GC coupled to a Trace DSQ II mass spectrometer (Thermo Fisher Scientific). Separation was achieved on a 30 m Zebron ZB-5 column 0.25 mm i.d., 0.25 µm film thickness (Phenomenex). The initial temperature of 50 °C was held constant for 5 min, followed by a ramp of 25 °C min^−1^ to a final temperature of 280 °C which was maintained for 5 min. The helium carrier gas flow rate was 1 ml min^−1^ and the injector temperature was set to 220 °C. Electron impact ionization (EI) was used at an ion source potential of 70 eV and a source temperature of 200 °C. Spectra were matched to the NIST (2.0) database using the Excalibur software. Additionally, standards of β-ionone and MHO (Sigma) were used.

Non-volatile reaction products were identified by LC-MS using a Dionex UltiMate 3000 UPLC coupled to a Q-Exactive mass spectrometer (Thermo Fisher Scientific). Sample separation was achieved with a Hypersil Gold C_18_ UPLC column (150 × 2.1 mm i.d., 1.9 µm) and the solvent system A, 0.05% (v/v) formic acid in H_2_O, and B, 0.05% (v/v) formic acid in acetonitrile. Initial conditions were 70% B for 1 min, followed by a gradient to 100% B within 4 min. The final conditions were maintained for 10 min, all at a flow rate of 0.5 ml min^−1^. Ionization of apocarotenoids was achieved with atmospheric pressure chemical ionization (APCI) and analyzed in the positive mode. Nitrogen was used as sheath and auxiliary gas, set to 20 and 10 arbitrary units, respectively. The vaporizer temperature was set to 350 °C and the capillary temperature was 320 °C. The spray voltage was set to 5 kV and the normalized collision energy (NCE) to 35 arbitrary units. For data analysis, the TraceFinder (3.1) software and authentic apocarotenoid standards were used.

## Results

### AtCCD4 cleaves cyclic carotenoids and apocarotenoids

Carotenoid-accumulating *E. coli* strains have frequently been used to test carotenoid-cleaving enzymes *in vivo* (von Lintig and Vogt, 2000; Booker *et al.*, 2004; Prado-Cabrero *et al.*, 2007; Frusciante *et al.*, 2014). However, only a limited number of carotenoid species can be produced in this system, and the stereospecificity of the reactions cannot be determined. We therefore resorted to *in vitro* assays utilizing an N-terminal thioredoxin–AtCCD4 fusion expressed in BL21 *E. coli* cells harboring the groEL-ES chaperone system (Alder *et al.*, 2008; Ilg *et al.*, 2009; Bruno *et al.*, 2014). Experiments were carried out with particle-free cell lysates and substrate-containing detergent micelles.

**Fig. 2.**
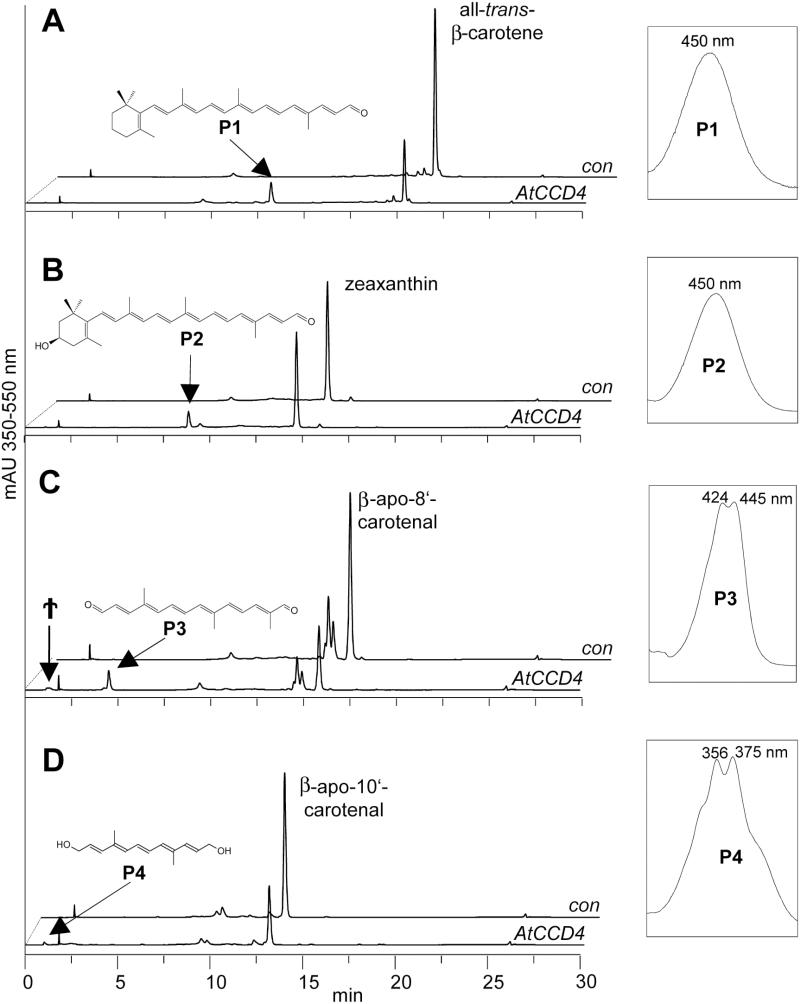
HPLC analysis of AtCCD4 activity. (A) Incubation of the enzyme with all-*trans*-β-carotene yielded β-apo-10'-carotenal (P1). (B) Cleavage of zeaxanthin yielded 3-OH-β-apo-10'-carotenal (P2). (C) Incubation with β-apo-8'-carotenal led to a C_17_ dialdehyde (P3) and traces of the corresponding C_17_ dialcohol (†). (D) β-Apo-10'-carotenal yielded the C_14_ dialcohol rosafluene (P4). UV/VIS spectra of the products are depicted in insets. The structures of the products are given. For structures of the substrates, see Supplementary Fig. 5. Analysis was performed using HPLC system 1.

First, we tested the cleavage of all-*trans*-β-carotene. The enzyme converted this substrate into product P1 that was identified as all-*trans*-β-apo-10'-carotenal by chromatographic and UV/VIS spectral comparison with the authentic reference (Fig. 2A). This product is consistent with a single cleavage, at either the C9–C10 or the C9'–C10' double bond. β-Ionone, the second cleavage product expected to arise upon the cleavage at this site, was identified by GC-MS analysis (Supplementary Fig. S1). Next, we investigated the stereospecificity of AtCCD4 by testing the conversion of 9-*cis*-β-carotene whose cleavage could liberate the SL precursor 9-*cis*-β-apo-10'-carotenal, which could indicate a contribution of AtCCD4 to SL biosynthesis. However, only marginal activity was observed, as witnessed by barely detectable traces of 9-*cis*-β-apo-10'-carotenal (Supplementary Fig. S2F). We also tested the cleavage of the asymmetric α-carotene that contains an ε-ionone and a β-ionone ring. This incubation led to a lower proportion of all-*trans*-β-apo-10'-carotenal (P1; Supplementary Fig. S2A) compared with all-*trans*-ε-apo-10'-carotenal (P5; Supplementary Fig. S2A), indicating preferred cleavage at the C9'–C10' double bond adjacent to the β-ionone ring. The enzyme also cleaved the asymmetric β,β-cryptoxanthin (carrying β-ionone and the 3-OH-β-ionone ring) at either the C9–C10 or the C9'–C10' double bond yielding traces of β-apo-10'-carotenal (P1) and higher amounts of 3-OH-β-apo-10'-carotenal (P2), indicating a preference for the double bond in the unhydroxylated moiety (Supplementary Fig. S2B). Though a previous report excluded the conversion of the dihydroxylated substrate zeaxanthin (Huang *et al.*, 2009), we detected a cleavage at the C9–C10 or the C9'–C10' double bond, yielding 3-OH-β-apo-10'-carotenal (P2; Fig. 2B). The dihydroxylated lutein, which carries a 3-OH-β- and -ε-ionone ring was converted—with a low efficiency—into 3-OH-β-apo-10'-carotenal and 3-OH-ε-apo-10'-carotenal (P2 and P6; Supplementary Fig. S2C), which arise by cleaving the C9–C10 or the C9'–C10' double bond. We also observed very low cleavage activity with all-*trans*-violaxanthin, which led to 5,6-epoxy-3-OH-β-apo-10'-carotenal (Supplementary Fig. S2D) tentatively identified based on the literature (Schwartz *et al.*, 2001). The 9-*cis* isomer of violaxanthin and all-*trans*-neoxanthin were not converted (Supplementary Fig. S2E, G). Taken together, AtCCD4 cleaved most of the C40-bicyclic carotenoids at the C9–C10 or the C9'–C10' double bond, with a preference for the double bond adjacent to unsubstituted β-ionone rings. Hydroxylation of both ionone rings led to a decrease in activity but did not affect the position of the cleavage site.

Next, we tested apocarotenoids with different chain lengths. AtCCD4 cleaved β-apo-8'-carotenal (C_30_) at the C9–C10 double bond, yielding a C_17_ dialdehyde (P3; Fig. 2C) and the corresponding C_17_ dialcohol (†; Fig. 2C) that probably derived from the dialdehyde product. The reduction of dialdehydes is frequently observed in assays utilizing *E. coli* lysate and is probably caused by an unspecific aldehyde dehydrogenase activity (Rodrigo *et al.*, 2013; Bruno *et al.*, 2015). GC-MS analysis confirmed the presence of the volatile β-ionone in these assays, which is expected to arise upon the formation of the C_17_-dialdehyde (Supplementary Fig. S1B). Consistently, the C_14_ dialcohol rosafluene was detected (P4; Fig. 2D) upon incubation with either β-apo-10'-carotenal (C_27_) or 3-OH-β-apo-10'-carotenal. Incubation with the shorter apocarotenal β-apo-12'-carotenal (C_25_) led only to traces of β-ionone. In conclusion, all apocarotenoids regardless of their chain length were cleaved at the C9–C10 double bond.

As shown above, AtCCD4 converted the C_40_ carotenoids β-carotene, β-cryptoxanthin, and zeaxanthin into β-apo-10'-carotenal and/or 3-OH-β-apo-10'-carotenal. However, this enzyme also cleaved its own products, β-apo-10'-carotenal and/or 3-OH-β-apo-10'-carotenal. To understand the biological function of AtCCD4, it is crucial to determine the preferred substrate, i.e. whether the enzyme favors C_40_-carotenoids or apocarotenoids. For this purpose, the assessment of standard parameters, *K*_M_ and *V*_max_, may be flawed due to the biphasic system used and to uncertainties concerning the equivalent micellar packing of the different substrate species in an enzyme-accessible form. Therefore, we have resorted to modeling of time course experiments in one homogeneous assay system in which bicyclic carotenoids are converted into monocyclic apocarotenoids that are again subjected to a further cleavage.

### Time course and modelling

**Fig. 3.**
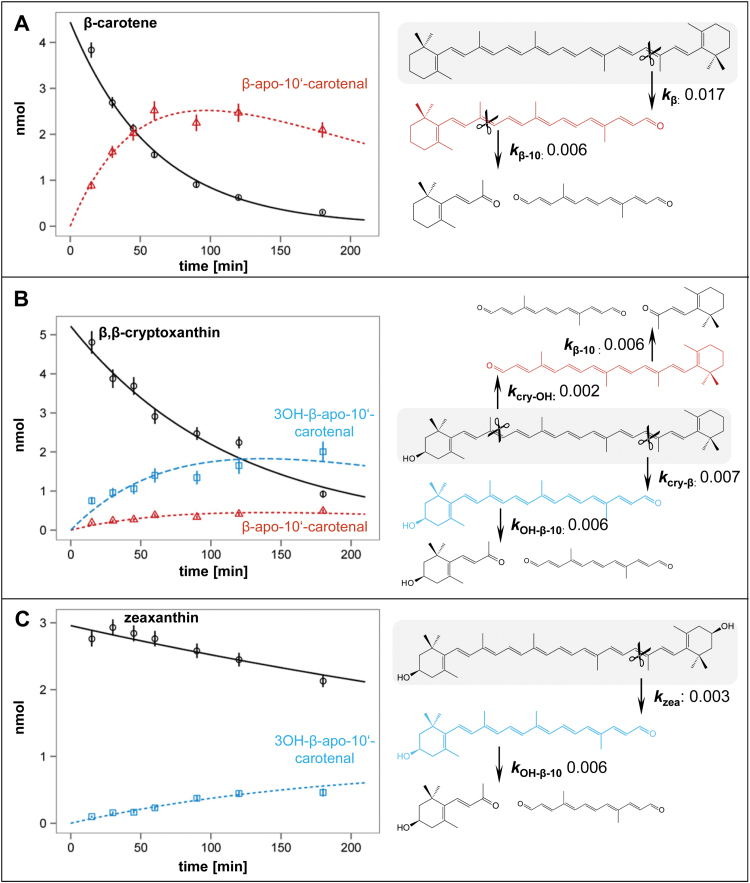
Time course and dynamic modeling of the AtCCD4 reaction. The conversion of three bicyclic carotenoids (shaded in gray) into the corresponding monocyclic C_27_ apocarotenoids and the subsequent cleavage of the latter are shown in (A–C). Note that the final products (3-OH)-β-ionone and the C_14_-dialdehyde were detected but could not be accurately quantified because of volatility, partition behavior, and instability. The symbols denote experimental data points and represent the average (± SEM) of 3–4 replicates. The line through the data points represents the model fit.

Dynamic modeling of AtCCD4 activity in time course experiments was carried out to determine rate constants for the primary (cleavage of bicyclic C_40_ carotenoids) and secondary cleavage reactions (cleavage of apocarotenoids) depicted in Fig. 3. Based on this, the rank order of bicyclic substrate decay is determined by the number of OH functions present, namely β-carotene (*k*_β_) >β-cryptoxanthin (*k*_cry-OH_+*k*_cry-β_) >zeaxanthin (*k*_zea_), the *k*-ratios being 1.0:1.9:5.7 (Fig. 3A–C). The asymmetric β-cryptoxanthin is preferentially cleaved at the C9–C10 site next to the unhydroxylated ionone (*k*_cry-β_=3.5 *k*_cry-OH_; Fig. 3B) corroborating that hydroxylation hinders cleavage.

It is conceivable that the two equivalent half sides of the symmetric β-carotene substrate can be cleaved by AtCCD4 with the same probability in terms of ‘half-side substrate recognition’. Instead of the expected *k*_β_≈2*k*_β-10_ (reflecting the double concentration of cleavable end-groups), the model supports *k*_β_=2.8 *k*_β-10_, indicating discrimination between the uncleaved bicyclic and the pre-cleaved monocyclic substrate. Furthermore, along these lines of thinking, β-ionone-containing substrate half-sides should be equivalent in bicyclic substrates, namely *k*_β_≈*k*_cry-β_. However, this is not the case either, so that the binding of the entire carotenoid molecule and the simultaneous recognition of both rings must be assumed. This notion is further supported by the fact that the model is consistent with a completely different behavior of the monocyclic C_27_ apocarotenoid substrates. Here, the preference for cleavage at unhydroxylated ionone rings is lost, the rate constants for the cleavage of β-apo-10'-carotenal and 3-OH-β-apo-10'-carotenal being very similar (*k*_β-0_≈*k*_OH-β-10_). This was experimentally verified by time course experiments using both apocarotenoids as substrates (Supplementary dataset S1). Consequently, losing one of the two binding determinants seemingly abolishes the differentiation observed with the bicyclic substrates.

As a result of hydroxylation, rate constants decreasd in bicylic substrates, whereas they remained relatively constant in monocyclic apocarotenoids. Opposing relative substrate preferences are consequently found. The absence of one ring can lower (*k*_β_/*k*_β-10_=2.8) or increase (*k*_*zea*_/*k*_OH-β-10_=0.4) the rate constants relative to those obtained for bicyclic symmetric substrates. This is further exemplified by the asymmetric (monohydroxylated) β-cryptoxanthin, where simultaneously rate constants relatively increase (*k*_cry-OH_/*k*_β-10_=0.3) or remain unchanged (*k*_cry-β_/*k*_OH-β-10_=1.0).

### CCD4 and/or CCD7 as potential generators of regulatory metabolites

AtCCD4 is thought to cleave the *cis*-configured phytoene desaturase (PDS) products phytofluene and/or ζ-carotene to generate regulatory molecules involved in leaf development (Avendaño-Vázquez *et al.*, 2014). Furthermore, genetic evidence indicated a role for an undefined CCD in cleaving *cis*-configured ζ-carotene desaturase (ZDS) desaturation intermediates to generate a feedback signaling compound regulating the transcription of the tomato *PSY1* (Kachanovsky *et al.*, 2012). Therefore, we examined four ζ-carotene isomers as potential AtCCD4 substrates. The canonical PDS product 9,15,9'-tri-*cis*-ζ-carotene as well as 9,9'-di-*cis*-ζ-carotene were used, the latter assuming a relevant contribution of the C15,C15' double bond-specific ζ-carotene-*cis*-*trans*-isomerase (Z-ISO) *in planta.* However, none of these substrates was cleaved (Supplementary Fig. S3A–D). Likewise, the enzyme did not convert the canonical PDS intermediate 9,15-di-*cis*-phytofluene (Supplementary Fig. S3E) or the isomers 15-*cis*-phytofluene and all-*trans*-phytofluene (Supplementary Fig. S3E). Thus, our *in vitro* data do not support the proposed involvement of CCD4 in leaf development.

To account also for a possible role in generating ζ-carotene- and lycopene-derived signals, we investigated AtCCD4 cleavage activity with the ZDS desaturation intermediate 7,9,9'-tri-*cis*-neurosporene (proneurosporene) and product 7,9,9',7'-tetra-*cis*-lycopene (prolycopene; Supplementary Fig. S3F, G). Assuming a relevant contribution of CRTISO, which can generate partially *trans*-isomerized neurosporene and lycopene (Isaacson *et al.*, 2004; Yu *et al.*, 2011), we also assayed 9'-*cis*-neurosporene (Supplementary Fig. S3H). However, the enzyme did not cleave any of these linear substrates (Supplementary Fig. 3A–H). Thus, our data are not in favor of an involvement of CCD4 in the regulation of *PSY* expression.

**Fig. 4.**
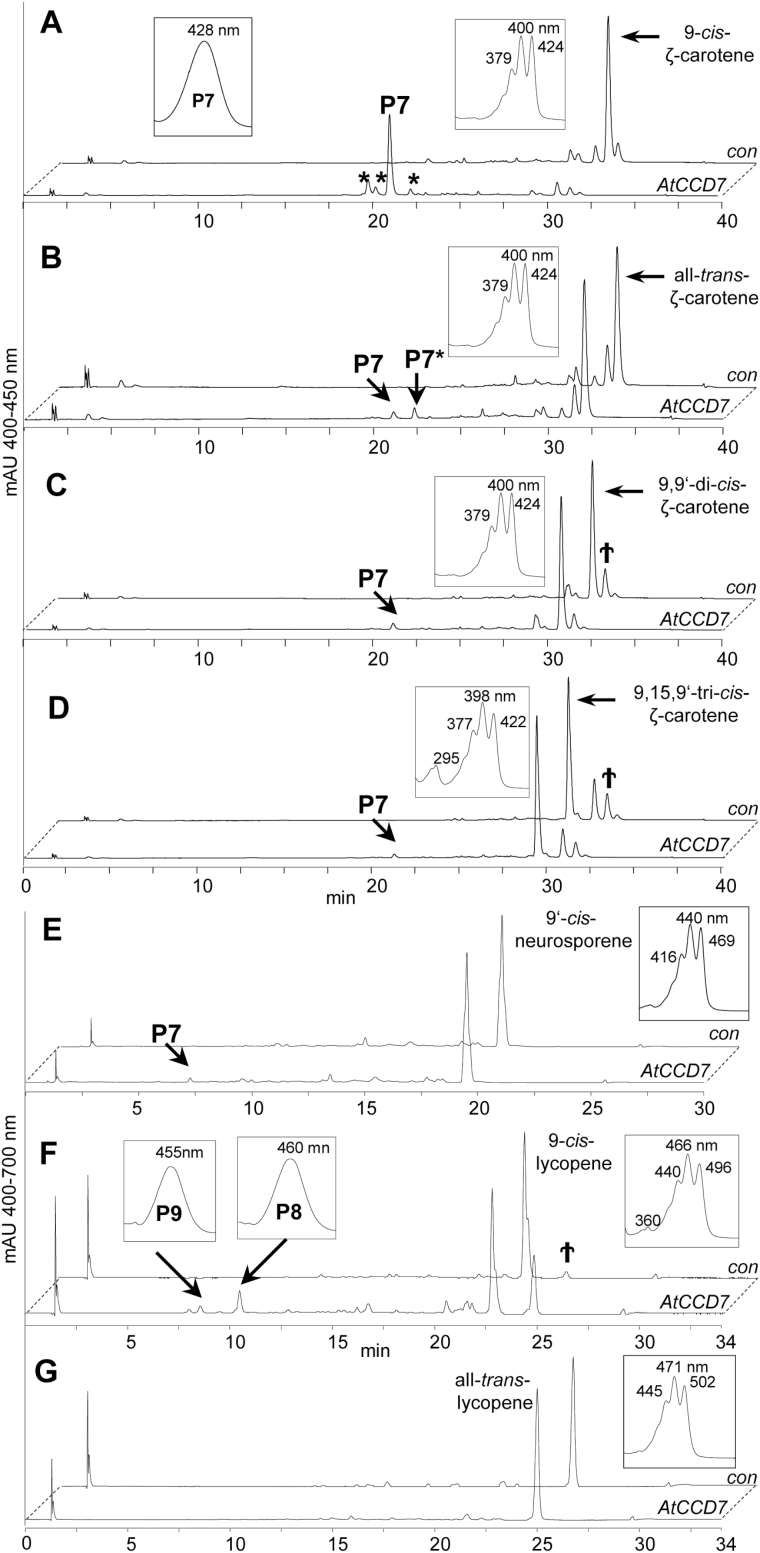
HPLC analysis of AtCCD7 activity with *cis*-configured carotene desaturation intermediates. (A) AtCCD7 cleaved 9-*cis*-ζ-carotene yielding P7, tentatively identified as 9-*cis*-ζ-apo-10'-carotenal. The minor peaks (asterisks) represent unspecific ζ-apo-10'-carotenal isomers—confirmed by isobaric masses (see Fig. 5A)—that arise upon sample processing. (B–D) Formation of traces of P7 upon incubation with other ζ-carotene isomers was due to minor cross-contamination with the 9-*cis* isomer (indicated by †). Product P7* corresponding to all-*trans*- ζ-apo-10'-carotenal was probably formed from P7 by unspecific isomerization. (E) AtCCD7 showed low cleavage activity with 9'-*cis*-neurosporene yielding P7, and (F) with 9-*cis*-lycopene yielding P8 and P9. P8 was identified by LC-MS analysis as all-*trans*-apo-10'-lycopenal, P9 as the putative 9-*cis*-apo-10'-lycopenal (see Fig. 6). The appearance of the all-*trans*-lycopene isomer upon incubation (indicated by †) and the formation of P8 are probably due to spontaneous *cis*-to-*trans* isomerization of 9-*cis*-lycopene and P8, respectively. (G) AtCCD7 did not convert all*-trans*-lycopene. UV/VIS spectra are shown as insets. For structures of the substrates, see Supplementary Figure 5. HPLC system 3 (A–D), HPLC system 1 (E), and HPLC system 2 (F, G) were used.

CCD7 enzymes are highly stereospecific, cleaving only 9-*cis*-configured substrates, such as 9-*cis*-β-carotene (Bruno *et al.*, 2014), which is produced from the all-*trans* species by the isomerase DWARF27 (Alder *et al.*, 2012; Bruno and Al-Babili, 2016). We therefore tested AtCCD7 as a candidate for the cleavage of *cis*-configured carotene desaturation intermediates. However, AtCCD7 did not convert any of the tri-*cis*-configured PDS or ZDS intermediates and products—9,15-di-*cis*-phytofluene, 9,15,9'-tri-*cis*-ζ-carotene, 7,9,9'-tri-*cis*-neurosporene, and 7,9,9',7-tetra-*cis*-lycopene. (Fig. 4D; Supplementary Fig. S4). Furthermore, the non-canonical isomers of phytofluene (9-*cis*, 15-*cis*, all-*trans*; Supplementary Fig. S4) were also not converted.

**Fig. 5.**
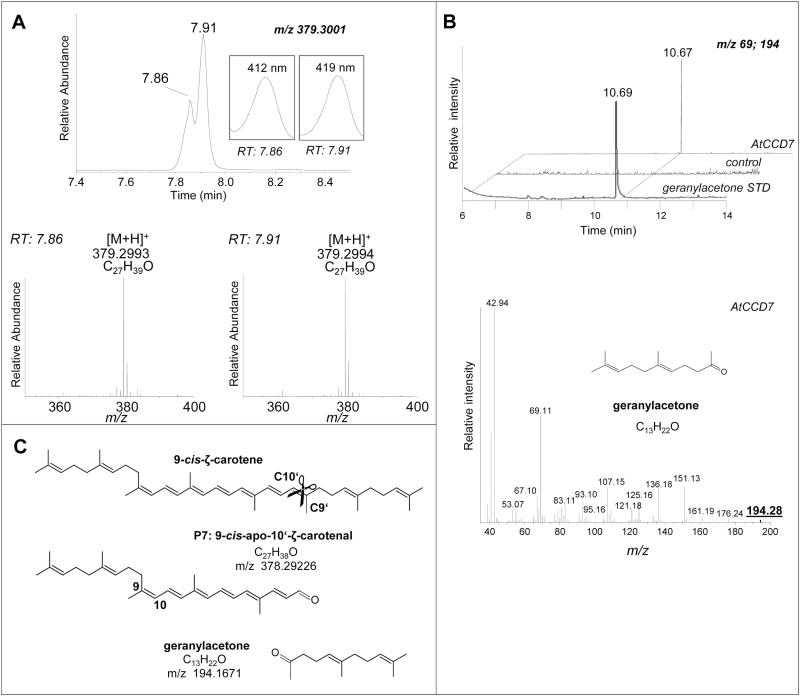
Identification of the 9-*cis*-ζ-carotene cleavage product. (A) The 9-*cis*-ζ-carotene cleavage product P7 (see Fig. 4A) was analyzed by LC-MS. AtCCD7 produced a putatively 9-*cis*-configured ζ-apo-10'-carotenal (7.91) accompanied by variable amounts of unspecific *cis*-*trans*-isomers (7.86), as shown by their isobaric masses. UV/VIS spectra of the products are depicted in insets. (B) GC-MS co-elution with the authentic reference (trace extracted at the indicated masses) and spectral comparison with the NIST 2.0 database identified geranylacetone as the volatile second cleavage product. (C) Schematic cleavage pattern of 9-*cis*-ζ-carotene. *m*/*z* denotes calculated isobaric masses.

**Fig. 6.**
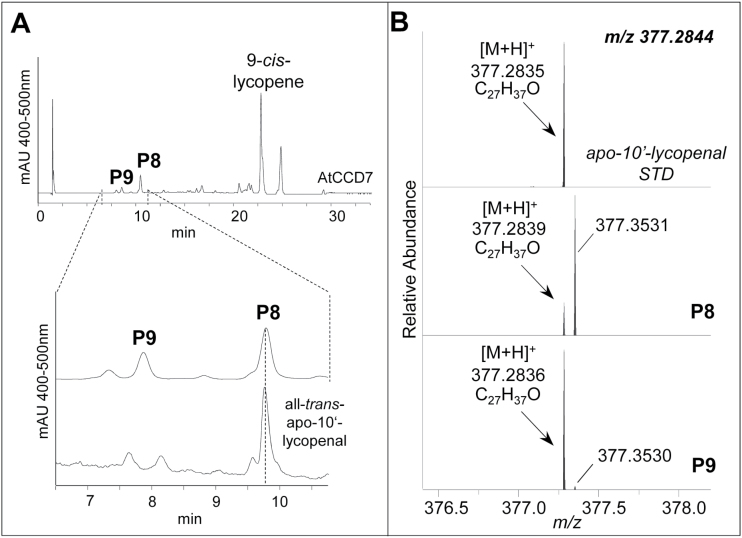
Identification of the 9-*cis*-lycopene AtCCD7 cleavage products. (A) The 9-*cis*-lycopene cleavage products P8 and P9 (see Fig. 4F) were analyzed by LC-MS. AtCCD7 produced a putatively 9-*cis*-configured apo-10'-lycopenal (P9) that was accompanied by the all-*trans* species identified based on an authentic reference (lower trace). (B) The isomeric nature is shown by the isobaric masses. For structures of the substrates, see Supplementary Fig. S5. HPLC system 2 was used for separation.

However, AtCCD7 cleaved the 9-*cis*-configured isomer of ζ-carotene (Fig. 4A–D) yielding the product P7 and small amounts of compounds with similar chromatographic and spectral characteristics (marked by asterisks in Fig. 4A). Subsequent LC-MS and GC-MS analyses revealed the formation of tentatively 9-*cis*-configured ζ-apo-10'-carotenal (P7, C_27_; Fig. 5A) and the volatile geranylacetone (C_13_; Fig. 5B). The other minor products detected in this incubation are probably *cis*/t*rans*-isomers of P7, which can be formed by non-enzymatic isomerization of P7. Incubation with a ζ-carotene sample, which contained all-*trans*-ζ-carotene as the major isomer, led to traces of P7. We also detected a minor peak (P7*) tentatively identified as the all-*trans*-isomer of ζ-apo-10'-carotenal. It can be assumed that P7* was formed from P7 by non-enzymatic isomerization. The formation of P7 trace amounts from the all-*trans*-ζ-carotene substrate is most probably due to unavoidable minor cross-contamination with the 9-*cis* species (indicated by † in Fig. 4C, D). Cleavage of 9-*cis-ζ*-carotene indicated an activity with 9'-*cis*-neurosporene that is half identical with ζ-carotene, while the second, more desaturated half corresponds to lycopene. To obtain this substrate, we used recombinant CRTISO that can produce the corresponding 9'-mono-*cis* isomer from the poly-*cis*-compounds proneurosporene and prolycopene (Isaacson *et al.*, 2004; Yu *et al.*, 2011). Incubation with AtCCD7 led to a single detectable product, most probably ζ-apo-10'-carotenal (P7), as verified by UV/VIS spectroscopic properties (Fig. 4E). This product suggests a cleavage in the more desaturated half side of neurosporene (Fig. 4E; Supplementary Fig. S5 XVIII). This half side should be *trans*-configured, based on CCD7’s strict stereospecificity (Bruno *et al.*, 2014), and on the preference of CRTISO (Yu *et al.*, 2011). Consequently the ζ-apo-10-carotenal yielded from 9'-*cis*-neurosporene is expected to carry a 9'-*cis* configuration, as shown in Fig. 5C. It needs to be noted that the conversion was much lower as compared with that obtained with 9-*cis-ζ*-carotene. (Fig. 4A). AtCCD7 showed weak activity with 9-*cis*-lycopene, yielding product P8 accompanied by traces of P9 (Fig. 4F), while all-*trans*-lycopene was not converted (Fig. 4G). LC-MS analysis and comparison with an all-*trans*-apo-10'-lycopenal standard identified both products as apo-10'-lycopenals, of which P8 represents the all-*trans*-species and P9 the 9-*cis* species (Fig. 6A, B). As shown in Fig. 4F, unspecific isomerization of 9-*cis*- to all-*trans*-lycopene in the presence of detergents and cleared cell lysates was commonly observed (indicated by † in Fig. 4F). In accordance with this observation and due to AtCCD7’s specificity for cleavage of the *trans*-configured C9–C10 double bond, we assume that P8 was formed from the sole cleavage product P9 by non-enzymatic isomerization.

## Discussion

### AtCCD4 cleavage site specificity and substrate preference

We have characterized the substrate, stereospecificity, and the cleavage pattern of AtCCD4. The results presented document AtCCD4 as an enzyme strictly cleaving the C9–C10 double bond. This regional specificity of cleavage was observed with bicyclic C_40_ carotenes and maintained with standard plant xanthophylls and derived (hydroxy-) apocarotenoids (Fig. 2A, B; Supplementary Fig. S2). The cleavage site is thus independent of hydroxyl functions in the C3 and C3' positions or on the presence of a second ionone ring. Cleavage site specificity was also unaffected by the chain length in (monocyclic) apocarotenoids (Fig. 2C, D). However, AtCCD4 requires the presence of at least one ionone ring for activity, since we did not observe any conversion of linear carotenes (Supplementary Fig. S3). Moreover, AtCCD4 is specific for all-*trans*-configured substrates and did not convert any mono- or poly-*cis*-configured substrates. This rules out a hypothetically possible direct contribution to ABA and SL biosynthesis (see AtCCD4 and regulatory molecules). Thus, AtCCD4 substrates need to be bicyclic or monocyclic and in the all-*trans* configuration to be converted.

Dynamic modeling of AtCCD4 time course experiments was carried out using different types of substrates, namely unhydroxylated (β-carotene), monohydroxylated (β-cryptoxanthin), and dihydroxylated (zeaxanthin) carotenoids, and the derived mono- and unhydroxylated apocarotenoids arising as secondary substrates upon primary cleavage. The resulting rate constants demonstrate that (i) unhydroxylated bicyclic C_40_ carotenes are preferred over C_27_ apocarotenoids; (ii) the presence of one or more OH groups in the C_40_ substrate reduces cleavage activity; and (iii) the latter does not apply to C_27_ apocarotenoids which, hydroxylated or not, were converted with very similar albeit low rate constants. It is worth noting that AtCCD1, an enzyme also targeting the C9–C10 double bond, prefers apocarotenoids over bicyclic carotenoids (Schmidt *et al.*, 2006; Ilg *et al.*, 2010). This difference is probably due to different biological functions and might be reflected by the cytoplasmatic localization of CCD1 that may contribute to carotenoid degradation *in planta* (Gonzalez-Jorge *et al.*, 2013; see the Introduction).

Upon incubation with violaxanthin, AtCCD4 showed only a marginal cleavage activity. However, two earlier publications on CCD4 mutants from potato (RNAi) and Arabidopsis (T-DNA insertion) reported on elevated total carotenoid levels, mainly of violaxanthin followed by antheraxanthin, and lutein (Campbell *et al.*, 2010; Gonzalez-Jorge *et al.*, 2013). This apparent contradiction can be explained by different substrate specificity *in planta* or by end-product accumulation that is caused by reducing the cleavage rate of the β-carotene precursor. Our *in vitro* data are consistent with the second option and, hence, they are in line with the *in vivo* data. However, it cannot be excluded that the enzyme’s preference is affected by the microenvironment *in planta*.

As deduced from the corresponding rate constants, AtCCD4 showed higher preference for C_40_ carotenoids than for apocarotenoids, indicating that the enzyme recognizes the entire C_40_ substrate and not only half sides. To gain insight into possible underlying structural elements allowing this discrimination, AtCCD4 structure predictions were carried out with I-TASSER (Zhang, 2008). The maize enzyme VP14 (NCED 3 in Arabidopsis), whose crystal structure has been elucidated (Messing *et al.*, 2010), was used as a comparator. AtCCD4 and VP14 share higher amino acid sequence homology (40% sequence identity) and structural similarity (TM score of 0.901) as compared with the other two CCDs for which structural information is available, SynACO (Kloer *et al.*, 2005) and RPE65 (Kiser *et al.*, 2009).

**Fig. 7.**
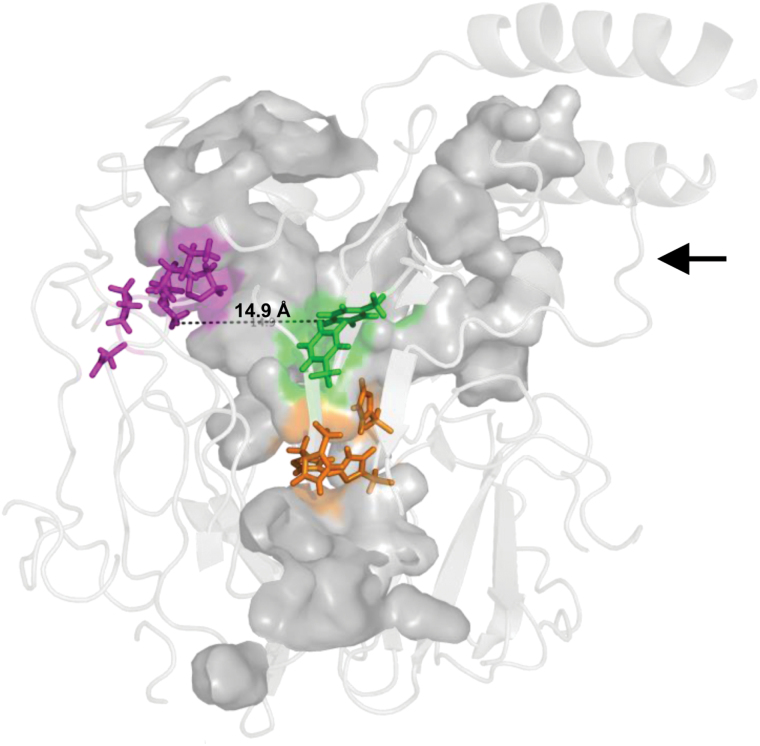
Predicted substrate cavity of AtCCD4. The substrate cavity is highlighted in gray and the conserved histidine residues co-ordinating Fe^2+^ are in orange. The conserved DPMPK motif on the back of the substrate cavity thought to restrict substrate penetration is shown in magenta. The ‘caging’ phenylalanine residues are highlighted in green. The distance from the rear of the cavity to the active center is ~15 Å, approximately the size of a β-ionone moiety, thus positioning the C9–C10 double bond for cleavage. For further explanations, see text.

Common CCD structural features (Sui *et al.*, 2013), including a hydrophobic surface patch to mediate association with membranes, are conserved (Supplementary Fig. S7A, B). Here, a cavity extends (arrowhead in Supplementary Fig. S7B) towards the active center lined with mainly hydrophobic residues. It is about 40 Å in length and hence well capable of accommodating entire C_40_ carotenoid substrates, as suggested with VP14 (Messing *et al.*, 2010). This supports our observations that are in favor of a recognition of the entire substrate molecule by AtCCD4. The cavity features a conserved motif at its back end (Messing *et al.*, 2010) consisting of two charged and three hydrophobic residues (D_499_PMPK_503_ in AtCCD4; pink in Fig. 7 and Supplementary Fig. S6) that might act as a ‘bumper’ for one ionone ring to restrict substrate penetration. These residues thus contribute to the positioning of the C9–C10 site relative to the active center that is formed by the conserved His298, His347, His412, and His590 (orange in Fig. 7 and Supplementary Fig. S6) co-ordinating Fe^2+^ on the central axis of the seven-blade β-propeller domain (blue in Supplementary Fig. S6A). Substrate positioning in VP14 is thought also to be aided by the three phenylalanine residues Phe171, Phe411, and Phe589, of which Phe411 is substituted by Ile411 in AtCCD4 (green in Fig. 7 and Supplementary Fig. S6) (Messing *et al.*, 2010). The position of the second ring appears less defined, at loop structures situated at the tunnel entrance. The structural identity of the aliphatic proportion in bicyclic substrates would consequently place a high selective significance on the ‘bumper’ site capable of accommodating unsubstituted β-ionone functions but also C3' hydroxylated functionss albeit with lower effectiveness. The distance to the reactive center of ~15 Å (dotted line, Fig. 7) might consequently determine regional specificity of cleavage.

VP14 is specific for 9-*cis*-configured epoxidated xanthophyll. This stereospecificity has been attributed to the presence of Val478, Ala478, or Ile478 in NCEDs, while CCDs carry phenylalanine or methionine in this position. With phenylalanine in this position, this would define AtCCD4 as a *trans*-specific CCD, a condition that is met by our data.

### AtCCD4 and regulatory molecules

For decades, evidence for retrograde regulation of carotenoid biosynthesis has been put forward, for instance in tomato (Giuliano *et al.*, 1993), maize (Bai *et al.*, 2009), daffodil (Al-Babili *et al.*, 1999), potato (Diretto *et al.*, 2010), and carrot (Arango *et al.*, 2014). The underlying mechanisms are far from being understood. However, it can be assumed that less lipophilic apocarotenoids that can be transported outside of the plastid play the role of signaling molecules, as known for ABA and SLs (Nambara and Marion-Poll, 2005; Raghavendra *et al.*, 2010; Al-Babili and Bouwmeester, 2015). As outlined (see the Introduction), data by Kachanovsky *et al.* (2012) and Avendaño-Vazquez *et al.* (2014) point to poly-*cis*-configured carotene desaturation intermediates as signal precursors, and the latter publication suggested a participation of AtCCD4 in the generation of such signals.

However, our *in vitro* studies show that AtCCD4 is incapable of cleaving any carotene desaturation intermediates. AtCCD4 cleaved neither the *cis*-ζ-carotene isomers 9,15,9'-tri-*cis*-, 9,9'-di-*cis*-, and 9-*cis*-ζ-carotene nor all-*trans*-ζ-carotene (Supplementary Fig. S3). Moreover, it did not convert any other linear carotene, such as phytofluene, neurosporene, or lycopene, irrespective of their stereo-configuration (Supplementary Fig. S3E–H). Our data are consistent with the report of Huang *et al.* (2009), which showed that AtCCD4 does not cleave ζ-carotene or lycopene in carotenoid-accumulating *E. coli* cells.

Our data do not support a contribution of AtCCD4 to the biosynthesis of the two known carotenoid-derived hormones ABA and SLs. In the case of SLs, this assumption is based on the stereospecificity of this enzyme that did not cleave 9-*cis*-β-carotene (Supplementary Fig. 2F), which would lead to 9-*cis*-apo-10'-carotenal, the SL biosynthesis intermediate formed by AtCCD7. Similarly, we did not observe any conversion of 9-*cis*-violaxanthin (Supplementary Fig. 2G), indicating that AtCCD4 neither contributes to nor directly interferes with ABA biosynthesis. This corroborates the previous observation that ABA levels in *ccd4* knock-down potatoes remain unaffected (Campbell *et al.*, 2010).

The enzyme produces all-*trans*-β-apo-10'-carotenal and -3-OH-β-apo-10'-carotenal from bicyclic carotenoids. Given that AtCCD4 mediates the synthesis of a signaling molecule, it might be speculated that this molecule is a derivative of these apocarotenoids. Indeed, all-*trans*-β-apo-10'-carotenal *in vitro* is a precursor of β-apo-13-carotenone (d'orenone) that is formed by CCD8 enzymes (Alder *et al.*, 2008). D'orenone has been shown to exert a regulatory function upon external application, affecting root hair growth in Arabidopsis (Schlicht *et al.*, 2008). Moreover, this compound triggers indole-3-acetic acid (IAA) synthesis in the ectomycorrhizal basidiomycete *Tricholoma vaccinum* and promotes lateral root growth in the host tree spruce (Wagner *et al.*, 2016). Taken together, our data do not support the hypothesis that AtCCD4 mediates the formation of signaling molecules from linear intermediates of the carotenoid biosynthesis pathway. However, it cannot be excluded that this enzyme plays a role in leaf morphogenesis or other developmental processes, for instance as a structural component of a complex regulating such processes. This assumption is based on the report of Naested *et al.* (2004) that shows the association of AtCCD4 with the protein VAR3 in a complex required for plastid development.

### A possible role for AtCCD7 in forming linear retrograde signals

Intriguingly, both known carotenoid-derived hormones, ABA and SLs, originate from 9-*cis*-configured carotenoids (Al-Babili and Bouwmeester, 2015). The 9-*cis* configuration is also present in the acyclic carotenes that are thought to be the precursor of new signaling molecule(s). All desaturation intermediates have a 9-*cis* configuration (as in phytofluene) or are *cis*-configured at both the C9–C10 and C9'–C10' double bond (Fig. 1; for structures, see Supplementary Fig. S5). Moreover, it can be assumed that plants also contain other 9-*cis*-configured carotenes, such as 9'-*cis*-neurosporene and 9-*cis*-lycopene, which do not act as pathway intermediates. 9'-*cis*-Neurosporene and 9-*cis*-lycopene can be enzymatically formed *in vitro* by the carotene isomerase CRTISO (Isaacson *et al.*, 2004; Yu *et al.*, 2011), a property that has been exploited in this study to generate these substrates.

Based on the reported specificity of AtCCD7 for 9-*cis*-configured bicyclic carotenoids (Bruno *et al.*, 2014), we tested the capability of this enzyme in cleaving desaturation intermediates and 9-*cis*-configured acyclic carotenes. The enzyme cleaved 9-*cis*-ζ-carotene, but did not show activity with any of the other ζ-carotene isomers tested. Thus, besides the strict specificity for 9-*cis*-configured carotenoids, AtCCD7 does not require the presence of an ionone ring structure for activity, in contrast to AtCCD4. Though to a much smaller extent, AtCCD7 also cleaved 9'-*cis*-neurosporene.

AtCCD7 maintained the regional specificity of the cleavage when incubated with acyclic carotenes. Taking the synthetic NMR-confirmed 9-*cis*-lycopene substrate as an example, cleavage of the *trans*-configured C9'–C10' double bond yielded 9-*cis*-apo-10'-lycopenal. The stereo-configuration of the product was demonstrated by its isobaric mass combined with different chromatographic behavior, compared with authentic all-*trans*-apo-10'-lycopenal (Fig. 6A, B). We conclude that AtCCD7 consequently formed 9-*cis*-ζ-apo-10'-carotenal from 9-*cis*-ζ-carotene and from 9'-*cis*-neurosporene. It remains to be shown whether the two AtCCD7 products 9-*cis*-ζ-apo-10'-carotenal and 9-*cis*-apo-10'-lycopenal are precursors of the signaling compounds that are thought to arise from carotene desaturation intermediates.

## Supplementary data

Supplementary data are available at *JXB* online.

Figure S1. GC-MS analysis of AtCCD4 volatile products.

Figure S2. HPLC analysis of AtCCD4 activity with different C_40_ xanthophylls and carotenes.

Figure S3. HPLC analysis of AtCCD4 activity with carotene desaturation intermediates.

Figure S4. HPLC analysis of AtCCD7 activity with carotene desaturation intermediates.

Figure S5. Structures of substrates cleaved by AtCCD4 and AtCCD7.

Figure S6. Sequence alignment of VP14, AtCCD4, and AtNCED3.

Figure S7. AtCCD4 3D model prediction.

Supplementary dataset S1. Modeling of AtCCD4 activity.

Supplementary Data

## References

[CIT0001] Al-Babili S Bouwmeester HJ 2015 Strigolactones, a novel carotenoid-derived plant hormone. Annual Review of Plant Biology 66, 161–186. 10.1146/annurev-arplant-043014-114759 25621512

[CIT0002] Al-Babili S Hartung W Kleinig H Beyer P 1999 CPTA modulates levels of carotenogenic proteins and their mRNAs and affects carotenoid and ABA content as well as chromoplast structure in *Narcissus pseudonarcissus* flowers. Plant Biology 1, 607–612.

[CIT0003] Alder A Holdermann I Beyer P Al-Babili S 2008 Carotenoid oxygenases involved in plant branching catalyse a highly specific conserved apocarotenoid cleavage reaction. Biochemical Journal 416, 289–96. 18637791 10.1042/BJ20080568

[CIT0004] Alder A Jamil M Marzorati M Bruno M Vermathen M Bigler P Ghisla S Bouwmeester H Beyer P Al-Babili S 2012 The path from β-carotene to carlactone, a strigolactone-like plant hormone. Science 335, 1348–1351. 22422982 10.1126/science.1218094

[CIT0005] Arango J Jourdan M Geoffriau E Beyer P Welsch R 2014 Carotene hydroxylase activity determines the levels of both α-carotene and total carotenoids in orange carrots. The Plant Cell 26, 2223–2233. 24858934 10.1105/tpc.113.122127 PMC4079379

[CIT0006] Auldridge ME McCarty DR Klee HJ 2006 Plant carotenoid cleavage oxygenases and their apocarotenoid products. Current Opinion in Plant Biology 9, 315–321. 16616608 10.1016/j.pbi.2006.03.005

[CIT0007] Avendaño-Vázquez A-O Cordoba E Llamas E 2014 An uncharacterized apocarotenoid-derived signal generated in ζ-carotene desaturase mutants regulates leaf development and the expression of chloroplast and nuclear genes in Arabidopsis. The Plant Cell 26, 2524–2537. 24907342 10.1105/tpc.114.123349 PMC4114949

[CIT0008] Bai L Kim E-H DellaPenna D Brutnell TP 2009 Novel lycopene epsilon cyclase activities in maize revealed through perturbation of carotenoid biosynthesis. The Plant Journal 59, 588–599. 19392686 10.1111/j.1365-313X.2009.03899.x

[CIT0009] Booker J Auldridge M Wills S Mccarty D Klee H Leyser O 2004 MAX3/CCD7 is a carotenoid cleavage dioxygenase required for the synthesis of a novel plant signaling molecule. Current Biology 14, 1232–1238. 15268852 10.1016/j.cub.2004.06.061

[CIT0010] Brandi F Bar E Mourgues F Horváth G Turcsi E Giuliano G Liverani A Tartarini S Lewinsohn E Rosati C 2011 Study of ‘Redhaven’ peach and its white-fleshed mutant suggests a key role of CCD4 carotenoid dioxygenase in carotenoid and norisoprenoid volatile metabolism. BMC Plant Biology 11, 11–24. 21269483 10.1186/1471-2229-11-24 PMC3045293

[CIT0011] Breitenbach J Sandmann G 2005 ζ-Carotene cis isomers as products and substrates in the plant poly-cis carotenoid biosynthetic pathway to lycopene. Planta 220, 785–793. 15503129 10.1007/s00425-004-1395-2

[CIT0012] Britton G Liaaen-Jensen S Pfander H, eds. 1995 Carotenoids, Vol 1B: Spectroscopy. Basel: Birkhäuser Verlag.

[CIT0013] Bruno M Al-Babili S 2016 On the substrate specificity of the rice strigolactone biosynthesis enzyme DWARF27. Planta 243, 1429–1440. 26945857 10.1007/s00425-016-2487-5

[CIT0014] Bruno M Beyer P Al-Babili S 2015 The potato carotenoid cleavage dioxygenase 4 catalyzes a single cleavage of β-ionone ring-containing carotenes and non-epoxidated xanthophylls. Archives of Biochemistry and Biophysics 572, 126–133. 25703194 10.1016/j.abb.2015.02.011

[CIT0015] Bruno M Hofmann M Vermathen M Alder A Beyer P Al-Babili S 2014 On the substrate- and stereospecificity of the plant carotenoid cleavage dioxygenase 7. FEBS Letters 588, 1802–1807. 24685691 10.1016/j.febslet.2014.03.041

[CIT0016] Campbell R Ducreux LJM Morris WL Morris J Suttle JC Ramsay G Bryan GJ Hedley PE Taylor M 2010 The metabolic and developmental roles of carotenoid cleavage dioxygenase4 from potato. Plant Physiology 154, 656–664. 20688977 10.1104/pp.110.158733 PMC2949026

[CIT0017] Carey L Clough JM Pattenden G 1983 Application of nuclear magnetic resonance spectroscopy in the stereochemical assignment of poly-Z-isomeric conjugated polyene isoprenoids. Journal of the Chemical Society, Perkin Transactions 1, 3005–3009.

[CIT0018] Clough JM Pattenden G 1979 Naturally occurring poly-cis carotenoids. Stereochemistry of poly-cis lycopene and its congeners in ‘Tangerine’ tomato fruits. Journal of the Chemical Society, Chemical Communications, 616–619.

[CIT0019] Diretto G Al-Babili S Tavazza R Scossa F Papacchioli V Migliore M Beyer P Giuliano G 2010 Transcriptional–metabolic networks in beta-carotene-enriched potato tubers: the long and winding road to the Golden phenotype. Plant Physiology 154, 899–912. 20671108 10.1104/pp.110.159368 PMC2949014

[CIT0020] Falchi R Vendramin E Zanon L Scalabrin S Cipriani G Verde I Vizzotto G Morgante M 2013 Three distinct mutational mechanisms acting on a single gene underpin the origin of yellow flesh in peach. The Plant Journal 76, 175–187. 23855972 10.1111/tpj.12283 PMC4223380

[CIT0021] Frusciante S Diretto G Bruno M 2014 Novel carotenoid cleavage dioxygenase catalyzes the first dedicated step in saffron crocin biosynthesis. Proceedings of the National Academy of Sciences, USA 111, 12246–12251. 10.1073/pnas.1404629111 PMC4143034 25097262

[CIT0022] Giuliano G Bartley GE Scolnik PA 1993 Regulation of carotenoid biosynthesis during tomato development. The Plant Cell 5, 379–387. 8485401 10.1105/tpc.5.4.379 PMC160278

[CIT0023] Gomez-Roldan V Fermas S Brewer PB 2008 Strigolactone inhibition of shoot branching. Nature 455, 189–195. 18690209 10.1038/nature07271

[CIT0024] Gonzalez-Jorge S Ha S-H Magallanes-Lundback M 2013 Carotenoid cleavage dioxygenase 4 is a negative regulator of β-carotene content in Arabidopsis seeds. The Plant Cell 25, 4812–4826. 24368792 10.1105/tpc.113.119677 PMC3903989

[CIT0025] Hagemann GE Sturgis JN Wagner E Robert B Beyer P Tadros MH 1996 The effects of the detergent LDAO on the carotenoid metabolism and growth of Rhodovulum sulfidophilum. Microbiological Research 151, 421–426.

[CIT0026] Huang F-C Molnár P Schwab W 2009 Cloning and functional characterization of carotenoid cleavage dioxygenase 4 genes. Journal of Experimental Botany 60, 3011–3022. 19436048 10.1093/jxb/erp137 PMC2718213

[CIT0027] Ilg A Beyer P Al-Babili S 2009 Characterization of the rice carotenoid cleavage dioxygenase 1 reveals a novel route for geranial biosynthesis. FEBS Journal 276, 736–747. 19120446 10.1111/j.1742-4658.2008.06820.x

[CIT0028] Ilg A Yu Q Schaub P Beyer P Al-Babili S 2010 Overexpression of the rice carotenoid cleavage dioxygenase 1 gene in Golden Rice endosperm suggests apocarotenoids as substrates *in planta* . Planta 232, 691–699. 20549230 10.1007/s00425-010-1205-y

[CIT0029] Ilg A Bruno M Beyer P Al-Babili S 2014 Tomato carotenoid cleavage dioxygenases 1A and 1B: relaxed double bond specificity leads to a plenitude of dialdehydes, mono-apocarotenoids and isoprenoid volatiles. FEBS Open Bio 4, 584–593. 10.1016/j.fob.2014.06.005 PMC4096678 25057464

[CIT0030] Isaacson T Ohad I Beyer P Hirschberg J 2004 Analysis in vitro of the enzyme CRTISO establishes a poly-cis-carotenoid biosynthesis pathway in plants. Plant Physiology 136, 4246–4255. 15557094 10.1104/pp.104.052092 PMC535854

[CIT0031] Kachanovsky DE Filler S Isaacson T Hirschberg J 2012 Epistasis in tomato color mutations involves regulation of phytoene synthase 1 expression by cis-carotenoids. Proceedings of the National Academy of Sciences, USA 109, 19021–19026. 10.1073/pnas.1214808109 PMC3503155 23112190

[CIT0032] Kiser PD Golczak M Lodowski DT Chance MR Palczewski K 2009 Crystal structure of native RPE65, the retinoid isomerase of the visual cycle. Proceedings of the National Academy of Sciences, USA 106, 17325–17330. 10.1073/pnas.0906600106 PMC2765077 19805034

[CIT0033] Kloer DP Ruch S Al-Babili S Beyer P Schulz GE 2005 The structure of a carotenoid oxygenase. Science 308, 267–269. 15821095 10.1126/science.1108965

[CIT0034] Ma G Zhang L Matsuta A Matsutani K Yamawaki K Yahata M Wahyudi A Motohashi R Kato M 2013 Enzymatic formation of β-citraurin from β-cryptoxanthin and zeaxanthin by carotenoid cleavage cioxygenase 4 in the flavedo of citrus fruit. Plant Physiology 163, 682–695. 23966550 10.1104/pp.113.223297 PMC3793050

[CIT0035] Messing SA Gabelli SB Echeverria I Vogel JT Guan JC Tan BC Klee HJ McCarty DR Amzel LM 2010 Structural insights into maize viviparous 14, a key enzyme in the biosynthesis of the phytohormone abscisic acid. The Plant Cell 22, 2970–2980. 20884803 10.1105/tpc.110.074815 PMC2965545

[CIT0036] Moise AR Al-Babili S Wurtzel ET 2014 Mechanistic aspects of carotenoid biosynthesis. Chemical Reviews 114, 164–193. 24175570 10.1021/cr400106y PMC3898671

[CIT0037] Naested H Holm A Jenkins T 2004 Arabidopsis VARIEGATED 3 encodes a chloroplast-targeted, zinc-finger protein required for chloroplast and palisade cell development. Journal of Cell Science 117, 4807–4818. 15340011 10.1242/jcs.01360

[CIT0038] Nambara E Marion-Poll A 2005 Abscisic acid biosynthesis and catabolism. Annual Review of Plant Biology 56, 165–185. 10.1146/annurev.arplant.56.032604.144046 15862093

[CIT0039] Nisar N Li L Shan L 2015 Carotenoid metabolism in plants. Molecular Plant 8, 68–82. 25578273 10.1016/j.molp.2014.12.007

[CIT0040] Nocedal J Wright SJ 1999 Numerical optimization. Berlin: Springer Science + Business Media, LLC.

[CIT0041] Ohmiya A Kishimoto S Aida R Yoshioka S Sumitomo K 2006 Carotenoid cleavage dioxygenase (CmCCD4a) contributes to white color formation in chrysanthemum petals. Plant Physiology 142, 1193–1201. 16980560 10.1104/pp.106.087130 PMC1630759

[CIT0042] Prado-Cabrero A Estrada AF Al-Babili S Avalos J 2007 Identification and biochemical characterization of a novel carotenoid oxygenase: elucidation of the cleavage step in the Fusarium carotenoid pathway. Molecular Microbiology 64, 448–460. 17493127 10.1111/j.1365-2958.2007.05665.x

[CIT0043] Raghavendra AS Gonugunta VK Christmann A Grill E 2010 ABA perception and signalling. Trends in Plant Science 15, 395–401. 20493758 10.1016/j.tplants.2010.04.006

[CIT0044] Raue A Kreutz C Maiwald T Bachmann J Schilling M Klingmüller U Timmer J 2009 Structural and practical identifiability analysis of partially observed dynamical models by exploiting the profile likelihood. Bioinformatics 25, 1923–1929. 19505944 10.1093/bioinformatics/btp358

[CIT0045] Rodrigo MJ Alquézar B Alós E Medina V Carmona L Bruno M Al-Babili S Zacarías L 2013 A novel carotenoid cleavage activity involved in the biosynthesis of Citrus fruit-specific apocarotenoid pigments. Journal of Experimental Botany 64, 4461–4478. 24006419 10.1093/jxb/ert260 PMC3808326

[CIT0046] Rubio A Rambla JL Santaella M Gómez MD Orzaez D Granell A Gómez-Gómez L 2008 Cytosolic and plastoglobule-targeted carotenoid dioxygenases from Crocus sativus are both involved in beta-ionone release. Journal of Biological Chemistry 283, 24816–24825. 18611853 10.1074/jbc.M804000200 PMC3259819

[CIT0047] Rubio-Moraga A Rambla JL Fernández-de-Carmen A Trapero-Mozos A Ahrazem O Orzáez D Granell A Gómez-Gómez L 2014 New target carotenoids for CCD4 enzymes are revealed with the characterization of a novel stress-induced carotenoid cleavage dioxygenase gene from Crocus sativus. Plant Molecular Biology 86, 555–569. 25204497 10.1007/s11103-014-0250-5

[CIT0048] Ruch S Beyer P Ernst H Al-Babili S 2005 Retinal biosynthesis in Eubacteria: in vitro characterization of a novel carotenoid oxygenase from Synechocystis sp. PCC 6803. Molecular Microbiology 55, 1015–1024. 15686550 10.1111/j.1365-2958.2004.04460.x

[CIT0049] Scherzinger D Al-Babili S 2008 In vitro characterization of a carotenoid cleavage dioxygenase from Nostoc sp. PCC 7120 reveals a novel cleavage pattern, cytosolic localization and induction by highlight. Molecular Microbiology 69, 231–244. 18485074 10.1111/j.1365-2958.2008.06282.x

[CIT0050] Schlicht M Samajová O Schachtschabel D Mancuso S Menzel D Boland W Baluska F 2008 D'orenone blocks polarized tip growth of root hairs by interfering with the PIN2-mediated auxin transport network in the root apex. The Plant Journal 55, 709–717. 18466302 10.1111/j.1365-313X.2008.03543.x

[CIT0051] Schmidt H Kurtzer R Eisenreich W Schwab W 2006 The carotenase AtCCD1 from Arabidopsis thaliana is a dioxygenase. Journal of Biological Chemistry 281, 9845–9851. 16459333 10.1074/jbc.M511668200

[CIT0052] Schwartz SH Qin X Zeevaart J 2001 Characterization of a novel carotenoid cleavage dioxygenase from plants. Journal of Biological Chemistry 276, 25208–25211. 11316814 10.1074/jbc.M102146200

[CIT0053] Schwartz SH Tan BC Gage DA Zeevaart JAD Mccarty DR 1997 Specific oxidative cleavage of carotenoids by VP14 of maize. Science 276, 1872–1874. 9188535 10.1126/science.276.5320.1872

[CIT0054] Seo M 2002 Complex regulation of ABA biosynthesis in plants. Trends in Plant Science 7, 41–48. 11804826 10.1016/s1360-1385(01)02187-2

[CIT0055] Soetaert K Petzoldt T Setzer RW 2010 Solving differential equations in *R*: package deSolve. Journal of Statistical Software 33, 1–25.20808728

[CIT0056] Sui X Kiser PD von Lintig J Palczewski K 2013 Structural basis of carotenoid cleavage: from bacteria to mammals. Archives of Biochemistry and Biophysics 539, 203–213. 23827316 10.1016/j.abb.2013.06.012 PMC3818509

[CIT0057] Umehara M Hanada A Yoshida S 2008 Inhibition of shoot branching by new terpenoid plant hormones. Nature 455, 195–200. 18690207 10.1038/nature07272

[CIT0058] Vogel JT Tan B Mccarty DR Klee HJ 2008 The carotenoid cleavage dioxygenase 1 enzyme has broad substrate specificity, cleaving multiple carotenoids at two different bond positions. Journal of Biological Chemistry 283, 11364–11373. 18285342 10.1074/jbc.M710106200

[CIT0059] von Lintig J Vogt K 2000 Filling the gap in vitamin A research. Molecular identification of an enzyme cleaving beta-carotene to retinal. Journal of Biological Chemistry 275, 11915–11920. 10766819 10.1074/jbc.275.16.11915

[CIT0060] Wagner K Krause K David A Kai M Jung E-M Sammer D Kniemeyer O Boland W Kothe E 2016 Influence of zygomycete-derived D'orenone on IAA signaling in Tricholoma–spruce ectomycorrhiza. Environmental Microbiology 18, 2470–2480. 26636983 10.1111/1462-2920.13160

[CIT0061] Walter MH Strack D 2011 Carotenoids and their cleavage products: biosynthesis and functions. Natural Product Reports 28, 663–692. 21321752 10.1039/c0np00036a

[CIT0062] Yu Q Ghisla S Hirschberg J Mann V Beyer P 2011 Plant carotene cis-trans isomerase CRTISO: a new member of the FAD(RED)-dependent flavoproteins catalyzing non-redox reactions. Journal of Biological Chemistry 286, 8666–8676. 21209101 10.1074/jbc.M110.208017 PMC3048748

[CIT0063] Zechmeister L 1962 Cis trans isomeric carotenoids, vitamins A and arylpolyenes. New York: Academic Press.

[CIT0064] Zhang Y 2008 I-TASSER server for protein 3D structure prediction. BMC Bioinformatics 9, 40. 18215316 10.1186/1471-2105-9-40 PMC2245901

[CIT0065] Zhu J-K 2002 Salt and drought stress signal transduction in plants. Annual Review of Plant Biology 53, 247–273. 10.1146/annurev.arplant.53.091401.143329 PMC3128348 12221975

