# Supplementary material for: Enzymatic study on AtCCD4 and AtCCD7 and their potential to form acyclic regulatory metabolites
Source: J Exp Bot. 2016 Oct 6;67(21):5993–6005. doi: 10.1093/jxb/erw356 (PMC5100015; doi:10.1093/jxb/erw356)
Supplement: Supplementary Data [file supp_67_21_5993__index.html]

Enzymatic study on AtCCD4 and AtCCD7 and their potential to form acyclic regulatory metabolites — Enzymatic study on AtCCD4 and AtCCD7 and their potential to form acyclic regulatory metabolites — Supplementary Data 

# Enzymatic study on AtCCD4 and AtCCD7 and their potential to form acyclic regulatory metabolites

## Supplementary Data

Data files

- supplementary\_figures\_S1\_S7.pdf - Supplementary Data
- Supplementary\_dataset\_S1.pdf - Supplementary Data
