# Supplementary material for: Enzymatic study on AtCCD4 and AtCCD7 and their potential to form acyclic regulatory metabolites
Source: J Exp Bot. 2016 Oct 6;67(21):5993–6005. doi: 10.1093/jxb/erw356 (PMC5100015; doi:10.1093/jxb/erw356)

Enzymatic study on AtCCD4 and AtCCD7 and their potential in forming carotenoid-derived retrograde signals

Mark Bruno, Julian Koschmieder, Florian Wuest, Patrick Schaub, Mirjam Fehling-Kaschek, Jens Timmer, Peter Beyer, and Salim Al-Babili

Supplementary Data

Figure S1. GC-MS analysis of AtCCD4 volatile products. (A) AtCCD4 cleaved β-carotene and β-apo-8'-carotenal, yielding a volatile compound with retention characteristics of authentic β-ionone that was not detected in the empty vector control cell lysates. (B) The identity of β-ionone was confirmed by its EI mass spectrum, indicating a cleavage of the C9, C10 double bond in both substrates. For structures of the substrates see Supplementary Figure S5.

Figure S2. HPLC analysis of AtCCD4 activity with different C_{40} xanthophylls and carotenes. (A) Incubation with α-carotene yielded ε-apo-10'-carotenal (P5) and traces of β-apo-10'-carotenal (P1), indicating preferred cleavage at the β-ionone adjacent C9-C10 site. (B) β,β-cryptoxanthin yielded 3-OH-β-apo-10'-carotenal (P2) and traces of β-apo-10'-carotenal (P1), indicating preferred cleavage in the un-hydroxylated moiety. (C) Incubation with lutein led to traces of 3-OH-ε-apo-10'-carotenal (P6) and of 3-OH-β-apo-10'-carotenal (P2). The predominant formation of P6 indicates preferred cleavage next to the 3-OH-β-ionone ring moiety. (D) All-*trans*-violaxanthin yielded traces of a putative 5,6-epoxy-3-OH-β-apo-10'-carotenal (asterisk). (E) No measurable activity was observed with all-*trans*-neoxanthin or the ABA precursor (G) 9-*cis*-violaxanthin. (F) 9-*cis*-β-carotene was not cleaved. The detection of P1 is due to a minor substrate contamination with all-*trans*-β-carotene (indicated by arrow head) that was preferentially cleaved despite of the large excess of the 9-*cis* species. This corroborates the specificity of the enzyme for all-*trans* configured substrates. Product UV/VIS spectra are given in (H). For structures of substrates, see Supplementary Figure S5. HPLC system 1 was used for separation.

Figure S3. HPLC analysis of AtCCD4 activity with carotene desaturation intermediates. (A-E) AtCCD4 did not convert any of the phytofluene or ζ-carotene isomers. (F-G) The more desaturated carotenes proneurosporene, its 9-*cis* isomer and prolycopene were also not converted. The UV/VIS spectra of the substrates are given. HPLC system 3 and 1 were used

in (A-G) and (H), respectively. For structures of substrates, see Supplementary Figure Fig. S5.

Figure S4. HPLC analysis of AtCCD7 activity with carotene desaturation intermediates. AtCCD7 did not convert any of the phytofluene isomers. Proneurosporene and prolycopene were not converted either. HPLC system 3 was used.

Figure S5. Structures of substrates cleaved by AtCCD4 and AtCCD7. (***I***) all-*trans*-β-carotene, (***II***) α-carotene, (***III***) β,β-cryptoxanthin, (***IV***) lutein, (***V***) zeaxanthin, (***VI***) all-*trans*-violaxanthin, (***VII***) all-*trans*-neoxanthin, (***VIII***) β-apo-8'-carotenal, (***IX***) β-apo-10'-carotenal, (***X***) 9-*cis*-β-carotene (***XI***), 9,15-di-*cis*-phytofluene, (***XII***) 9-*cis*-phytofluene, (***XIII***) 9,15,9'-tri-*cis*-ζ-carotene, (***XIV***) 9,9'-di-*cis*-ζ-carotene, (***XV***) 9-*cis*-ζ-carotene, (***XVI***) all-*trans*-ζ-carotene, (***XVII***) 7,9,9'-tri-*cis*-neurosporene, (***XVIII***) 9'-*cis*-neurosporene, (***XIX***) 7,9,9',7'-tetra-*cis*-lycopene (prolycopene), (***XX***) 7,9-di-*cis*-lycopene, (***XXI***) all-*trans*-lycopene, (***XXII***) 9-*cis*-lycopene. Scissors indicate cleavage positions at the C9-C10 or C9'-C10' double bonds. The figure shows AtCCD7 cleavage sites in acyclic carotenes. The specific AtCCD4/AtCCD7 activities are indicated. Scissors in light grey indicate minor cleavage activities.

Figure S6. Sequence alignment of VP14, AtCCD4 and AtNCED3. Black background indicates sequence identity; squirreled lines indicate α-helices and arrows β-sheets. Conserved histidine residues (His_{298}, His_{347}, His_{412} His_{590}) are highlighted in orange. Conserved Glu residues (Glu_{477} is substituted by Asp in AtCCD4) shielding the four His residues are highlighted in red. Phe residues constituting the substrate cage are highlighted in green (note that in AtCCD4 Phe_{411} is substituted by Ile). Highlighted in blue are Val_{478} in VP14 and Phe_{478} in AtCCD4 possibly acting as a selector for *trans*- or *cis*-configured substrates. Pink box with circles indicates the site (EPWPK in VP14; DPMPK in AtCCD4) on the rear side of the substrate restricting substrate penetration. Clustal ω was used for the alignment and the figure prepared with ESPript (Gouet et al., 1999). Acc. No. *Zea mays* VP14 (AAB62181.2), *Arabidopsis thaliana* CCD4 (NP_193652.1) and *Arabidopsis thaliana* NCED3 (Q9LRR7.1).

Figure S7. AtCCD4 3D model prediction. VP14 structure from maize (3npe) showed highest TM-score (0.901) (A) Highlighted in red are the α-helices forming the dome structure and constituting to the hydrophobic patch. The β-propeller sheets are highlighted in blue. The conserved His residues coordinating Fe^{2+} are in orange, and the conserved DPMPK motif on the rear side of the substrate cavity is given in pink. (B) Electrochemical surface potential of AtCCD4. The protein orientation is as in (A). Surface potentials (calculated for pH7.0) are color-coded as indicated. The hydrophobic patch mediating membrane association is visible on top of the protein. The substrate cavity entrance is indicated by an arrowhead. The model was created with I-TASSER (Yang *et al.*, 2015; Zhang, 2008) and visualized using Pymol.

Supplementary S1

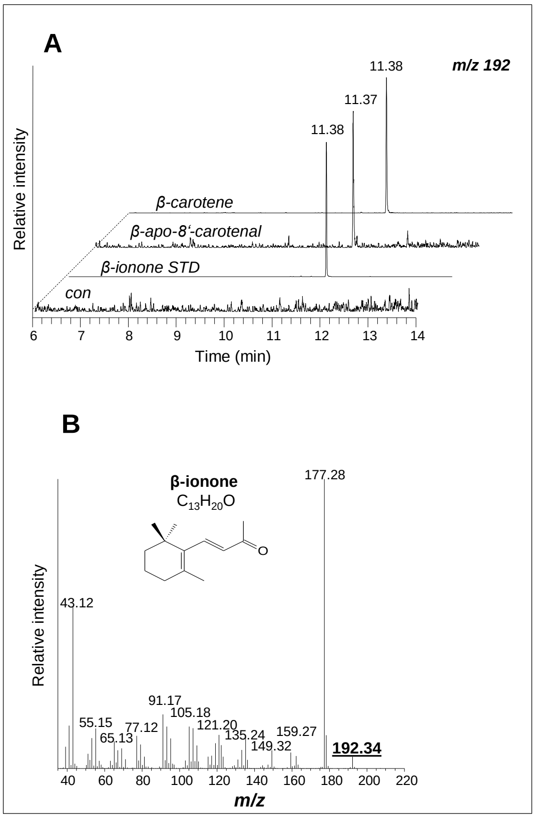

Supplementary S2

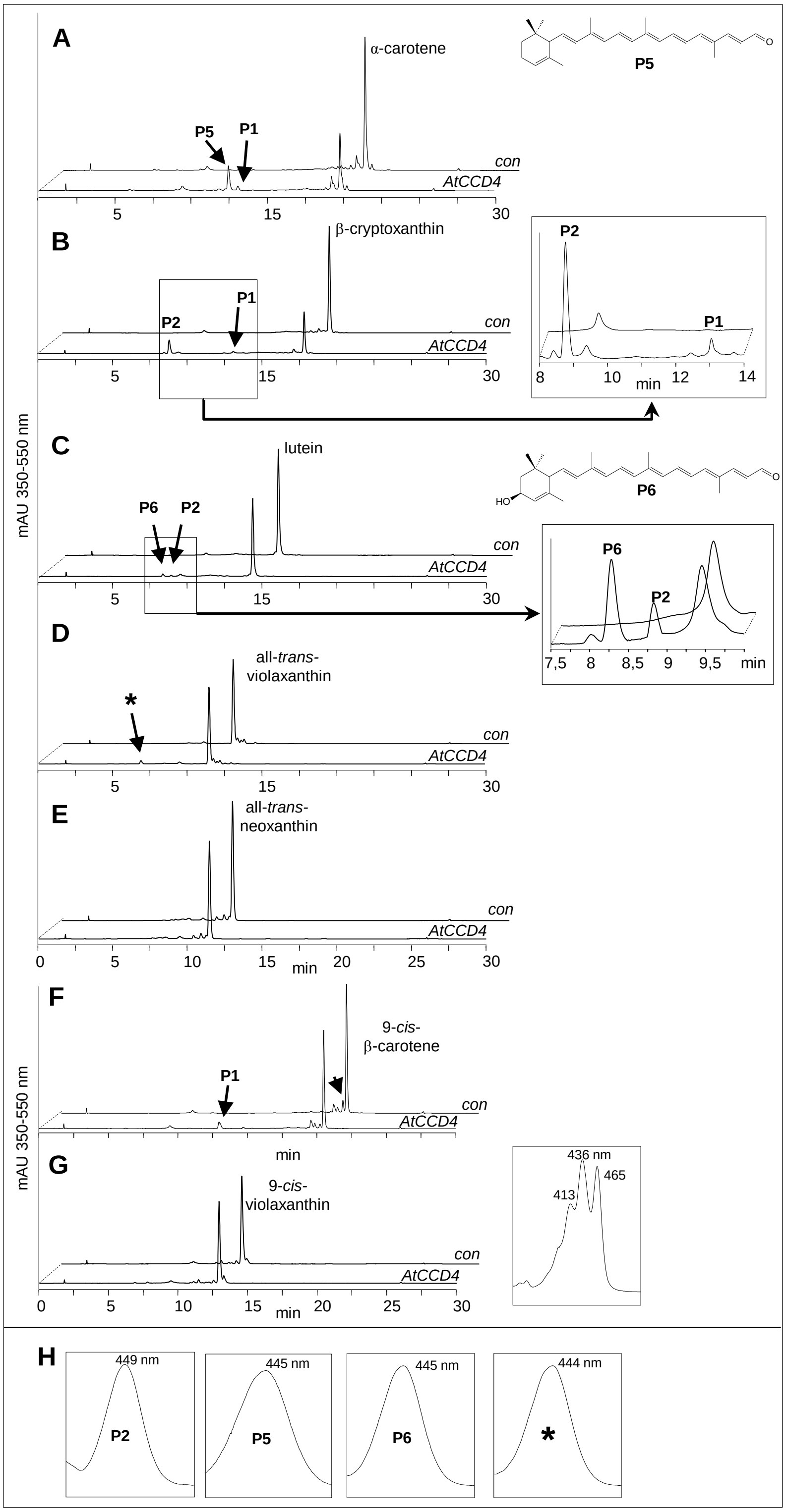

Supplementary S3

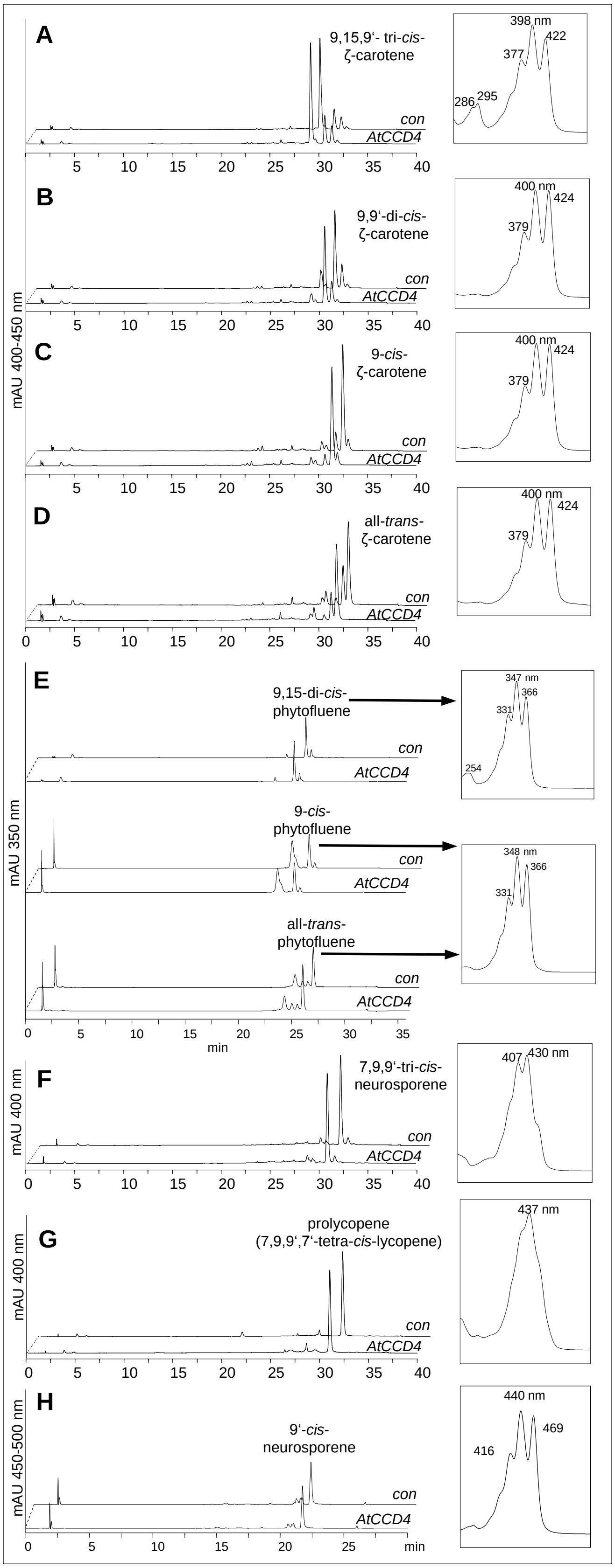

Supplementary S4

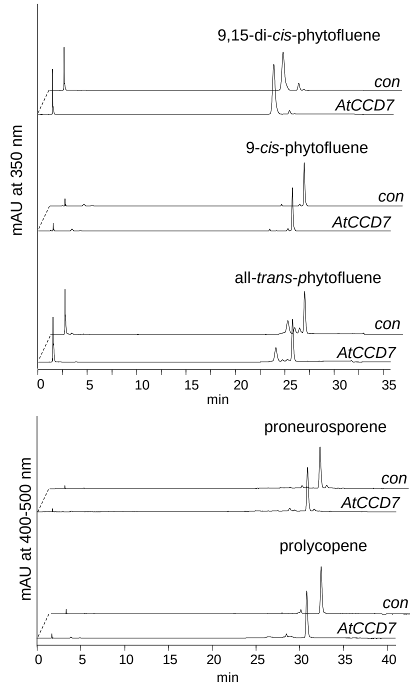

Supplementary Fig. S5

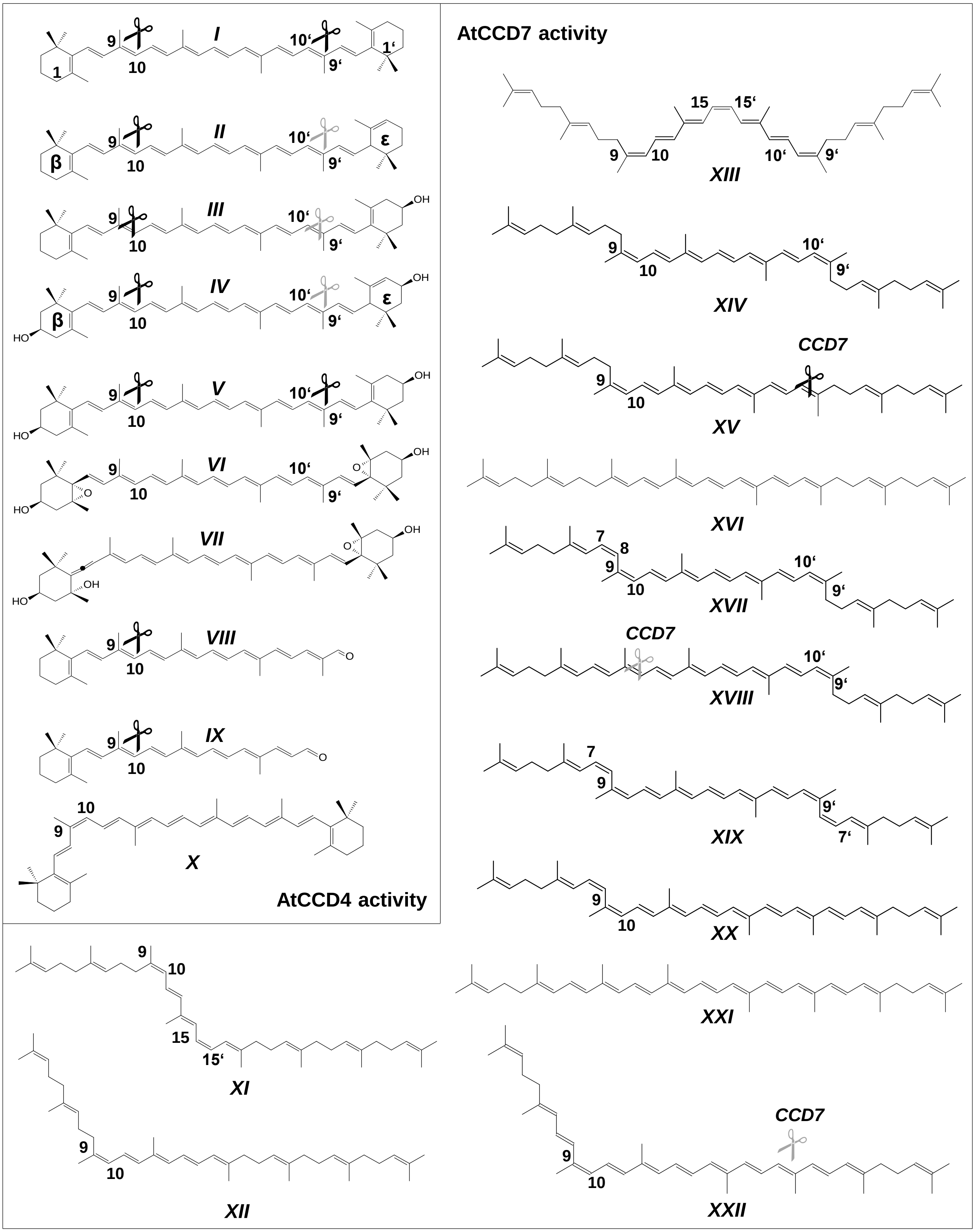

Supplementary S6

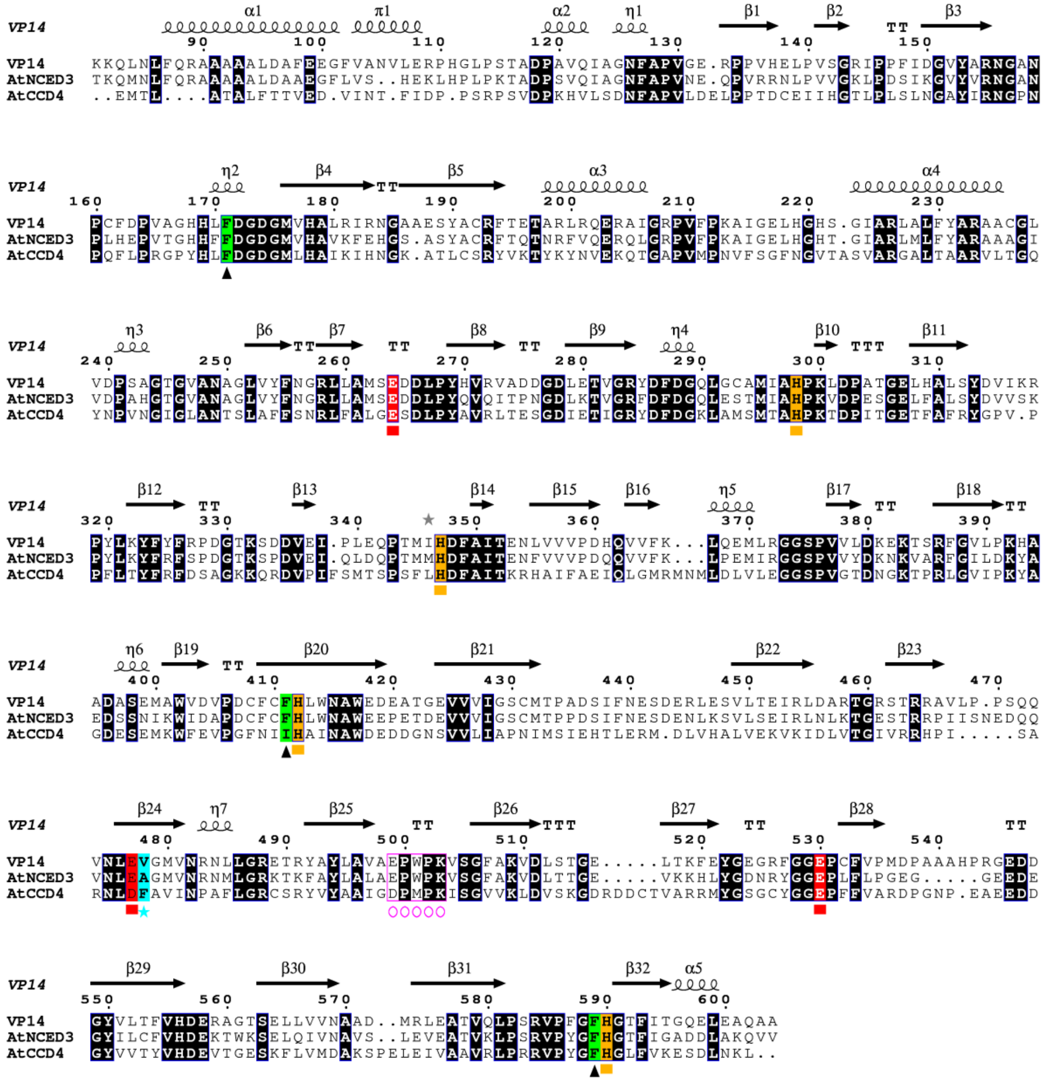

Supplementary S7

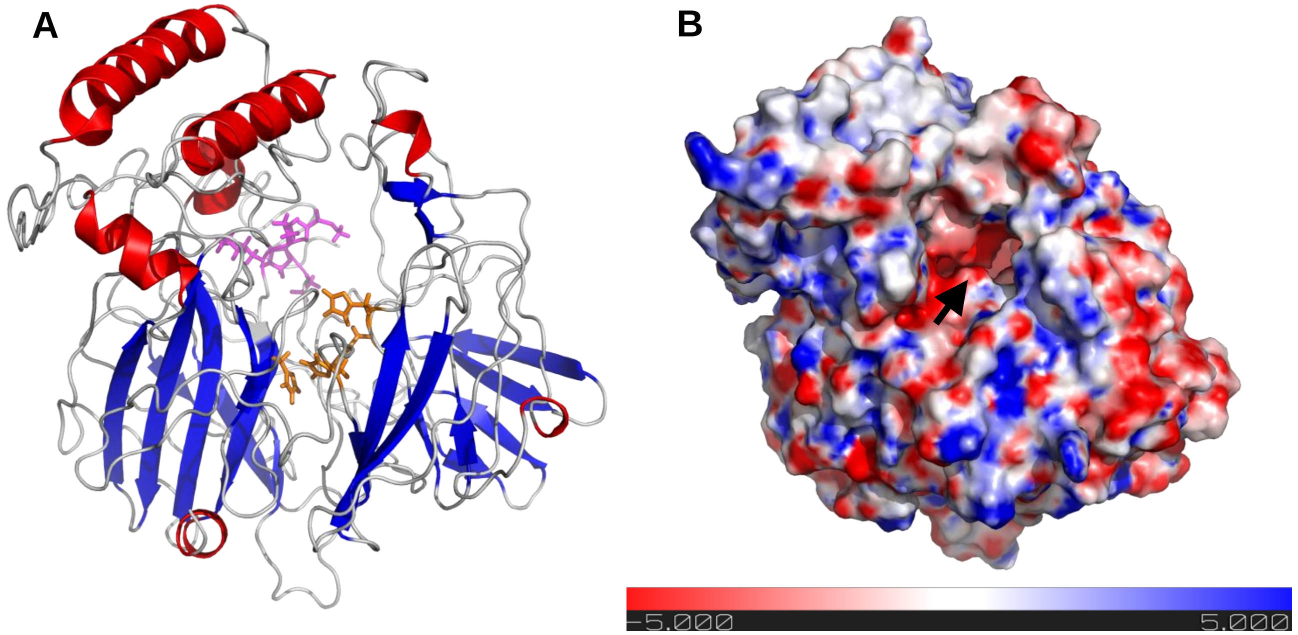

Supplement: Supplementary Data [file supp_erw356_supplementary_figures_S1_S7.pdf]
