# Supplementary material for: Enzymatic study on AtCCD4 and AtCCD7 and their potential to form acyclic regulatory metabolites
Source: J Exp Bot. 2016 Oct 6;67(21):5993–6005. doi: 10.1093/jxb/erw356 (PMC5100015; doi:10.1093/jxb/erw356)
Supplement: Supplementary Data [file supp_erw356_Supplementary_dataset_S1.pdf]

Supplementary dataset S1

The model consists of the ODEs, given in *Dynamic modelling and data preprocessing*, for the states β, cry, zea, β-10, OH-β-10, β-io, and OH-β-io. All states, but β-io and OH-β-io, are observables as they can be directly measured. All state initial values are zero except for the one of the initially added substrate. The datasets (constituting one time course each) with the corresponding non-zero initial value parameters and the measured quantities (observables) are listed in Table 1.

Table 1: List of datasets used for modelling.

Dataset	Initial value parameter	Observables
β_1	ini_β1	β, β-10
β_2	ini_β2	β, β-10
cry	ini_cry	cry, β-10, OH-β-10
zea	ini_zea	zea, OH-β-10
β-10	ini_β-10	β-10
OH-β-10	ini_OH-β-10	OH-β-10

A parameter transformation is used to set the initial β parameters to different values for the β_1 and β_2 condition. The β_2 and zea datasets were produced with a different enzyme preparation compared to the other conditions. All rate parameters of these two datasets are therefore multiplied with a conversion factor, which is a free parameter being determined jointly with the other rate parameters by maximum-likelihood estimation. In addition, all parameters are transformed to log-scale for the parameter estimation.

Parameters are estimated by a simultaneous fit of all conditions, yielding the values listed in Table 2 for the rate constants.

Table 2: Values and standard errors of the rate constant parameters.

Parameter	Value		Σ
k_{β}	0.0166	±	0.0005
$k_{\beta\text{-}10}$	0.0058	±	0.0005
$k_{cry\text{-}OH}$	0.0017	±	0.0001
$k_{cry\text{-}\beta}$	0.0070	±	0.0004
$k_{OH\text{-}\beta\text{-}10}$	0.0061	±	0.0010
k_{zea}	0.0031	±	0.0002

The model prediction with corresponding data is shown in Figure 3 of the main text for all conditions but β-10, OH-β-10 and β_2, which are shown in Figure1 and 2. The decay parameters of β-10 and OH-β-10, $k_{\beta\text{-}10}$ and $k_{OH\text{-}\beta\text{-}10}$, are similar which can be seen in the logarithmic representation of model prediction and data in Figure 1. The reduced enzyme activity in the β_2 dataset compared to the β_1 dataset is visible in Figure 2.

Confidence intervals of the estimated parameters are determined by the profile-likelihood method. Based on the likelihood profiles (continuous lines), confidence intervals are obtained by projection of the points of intersection with the corresponding confidence level thresholds (dashed lines). Profiles of all estimated parameters are shown in Figures 3 and 4.

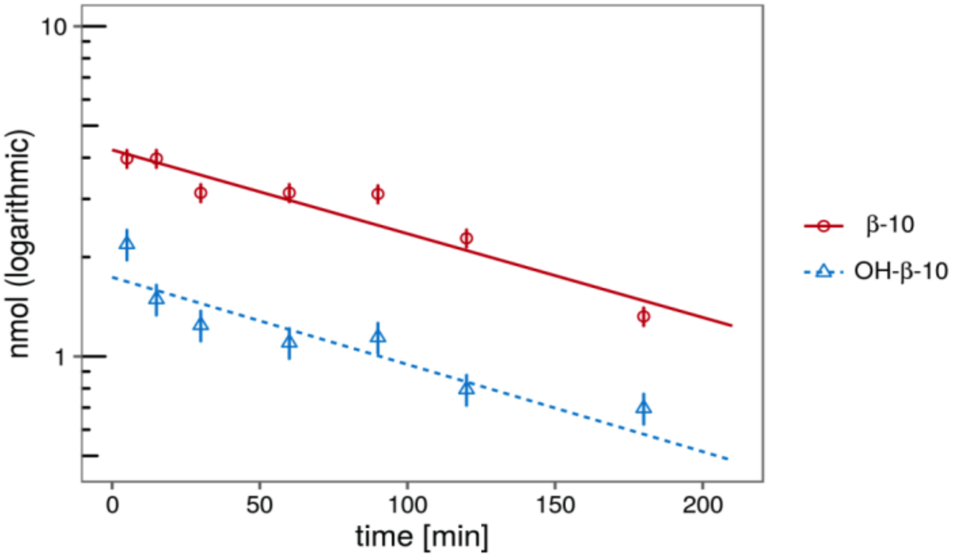

Figure 1: Model prediction and data for the β-10 and OH-β-10 datasets.

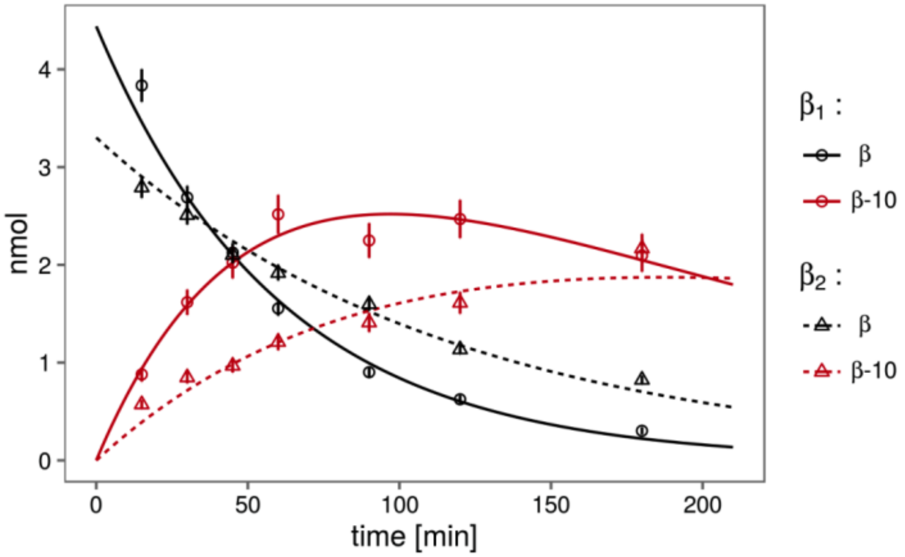

Figure 2: Model prediction and data for the β_1 and β_2 datasets.

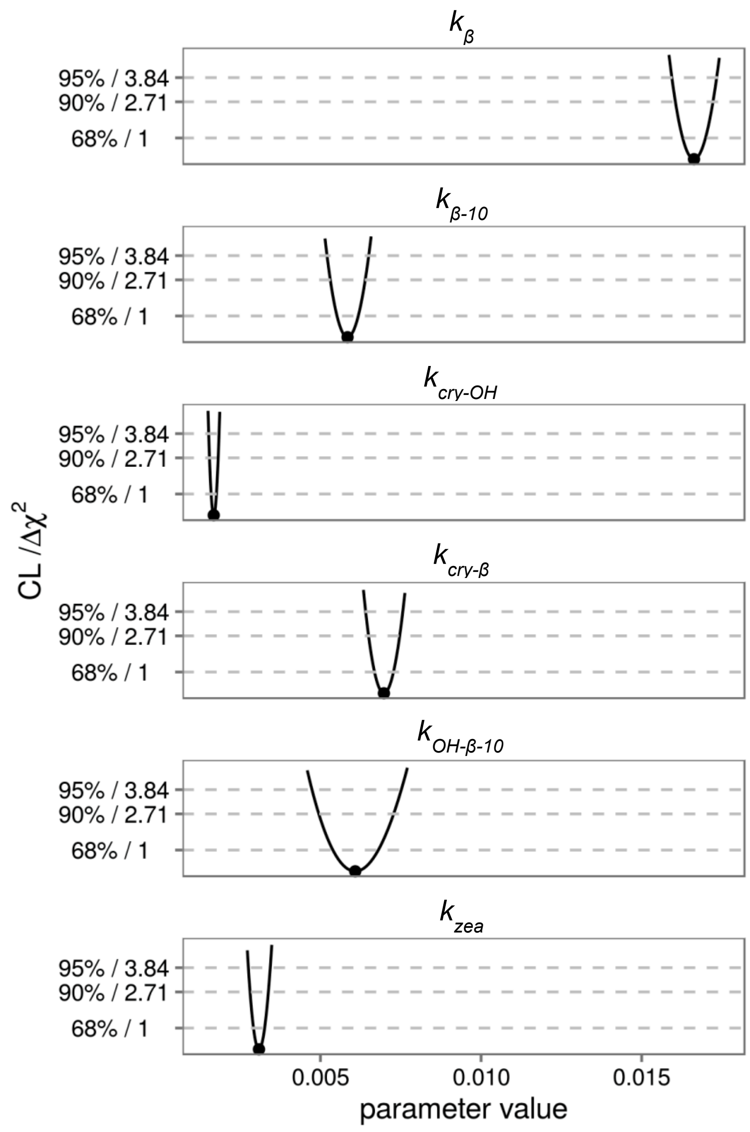

Figure 3: Profiles of the rate constant parameters.

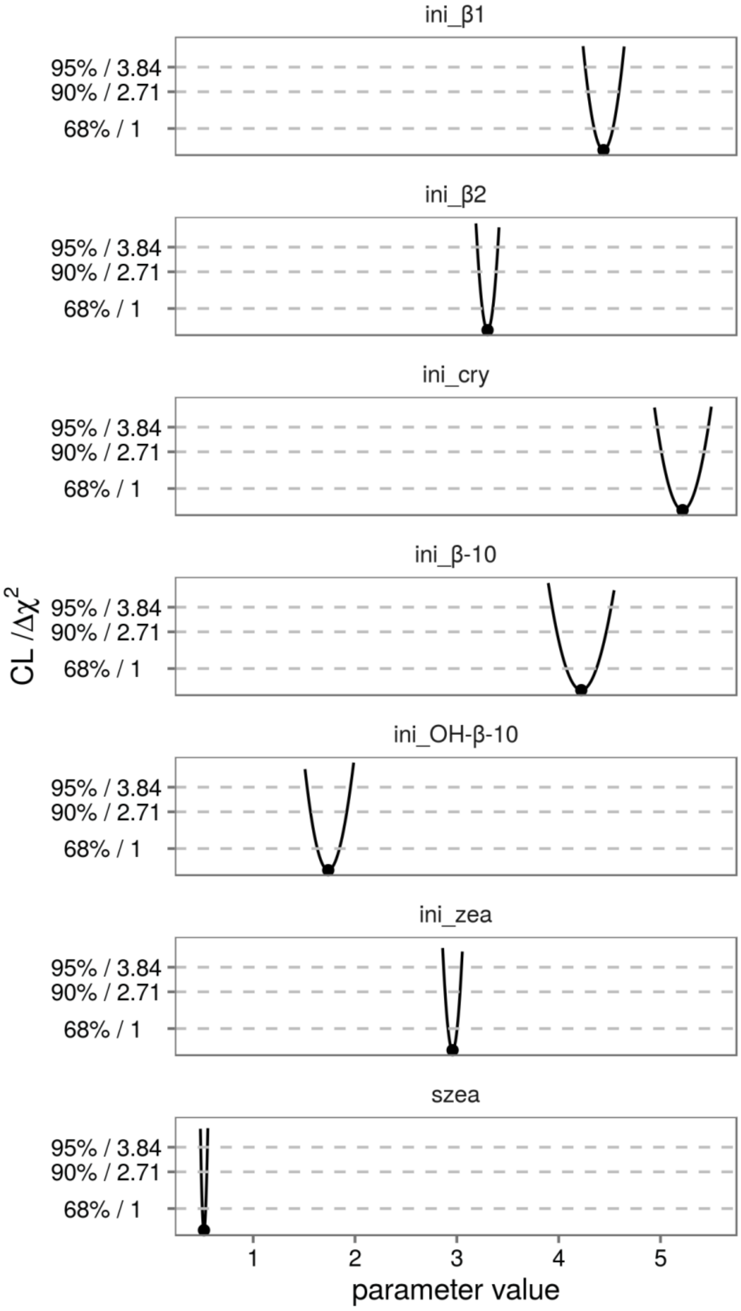

Figure 4: Profiles of the initial value parameters and the conversion factor szea.
